# The flagellin of candidate live biotherapeutic *Enterococcus gallinarum* MRx0518 is a potent immunostimulant

**DOI:** 10.1038/s41598-018-36926-8

**Published:** 2019-01-28

**Authors:** Delphine L. Lauté-Caly, Emma J. Raftis, Philip Cowie, Emma Hennessy, Amy Holt, D. Alessio Panzica, Christina Sparre, Beverley Minter, Eline Stroobach, Imke E. Mulder

**Affiliations:** 4D Pharma Research Ltd, Life Science Innovation Building, Cornhill Road, Aberdeen, AB25 2ZS United Kingdom

## Abstract

Many links between gut microbiota and disease development have been established in recent years, with particular bacterial strains emerging as potential therapeutics rather than causative agents. In this study we describe the immunostimulatory properties of *Enterococcus gallinarum* MRx0518, a candidate live biotherapeutic with proven anti-tumorigenic efficacy. Here we demonstrate that strain MRx0518 elicits a strong pro-inflammatory response in key components of the innate immune system but also in intestinal epithelial cells. Using a flagellin knock-out derivative and purified recombinant protein, MRx0518 flagellin was shown to be a TLR5 and NF-κB activator in reporter cells and an inducer of IL-8 production by HT29-MTX cells. *E. gallinarum* flagellin proteins display a high level of sequence diversity and the flagellin produced by MRx0518 was shown to be more potent than flagellin from *E. gallinarum* DSM100110. Collectively, these data infer that flagellin may play a role in the therapeutic properties of *E. gallinarum* MRx0518.

## Introduction

*Enterococcus gallinarum* is a commensal Gram-positive species that sits within the *Enterococcus* *casseliflavus* clade of the *Enterococcus* 16 S rRNA phylogenic tree^1,2^. *E. gallinarum* and *E. casseliflavus* species are closely related, sharing over 99.8% nucleotide identity between their 16S rRNA genes^3^. *E. gallinarum* and *E. casseliflavus* are the only enterococcal species that are described as motile^4,5^ and unlike other members of the *Enterococcus* genus, they are infrequently linked with nosocomial infections^6,7^.

In recent years, the role of the intestinal microbiota in cancer has received increasing attention because of its importance for immunotherapy efficacy (see review by Kroemer *et al*.^8^). Species in the *Enterococcus* genus have been identified as having potential uses in the growing field of oncobiotics^9,10^. Specifically, *Enterococcus hirae* has been shown to enhance cyclophosphamide efficacy by stimulating an anti-tumorigenic adaptive immune response following translocation to secondary lymphoid organs^9^. Routy *et al*. have also shown that *E. gallinarum* and several other enterococcal species were relatively overly abundant in patients who responded to immune checkpoint inhibitors (ICI)^11^. We have recently demonstrated that *E. gallinarum* MRx0518, a commensal strain that was isolated from a healthy human gut produces robust anti-tumorigenic effects after prophylactic oral dosing in murine models of breast, lung and renal carcinomas^12^.

Flagellin from certain bacterial species are considered to be immunostimulatory and have also been exploited for their anti-tumorigenic and radioprotective potential (recently reviewed by Hajam *et al*.^13^). A *Vibrio vulnificus* flagellin expressed in an attenuated strain of *Salmonella* Typhimurium demonstrated tumour suppressive effects and decreased metastasis in murine models of orthotropic human colon cancer, when delivered intravenously^14^. Additionally, a *Salmonella enterica* flagellin derivative (CBLB502), is under investigation for the treatment of patients with advanced solid tumours^15^. Subcutaneous injection of this flagellin protein reduced tumour growth in a murine model of T-cell lymphoma through induction of pro-inflammatory cytokines and activation of cytotoxic lymphocytes^16^.

Flagellin is a well-studied microbe-associated molecular pattern that is recognized by the transmembrane Toll-like receptor 5 (TLR5), which regulates the induction of downstream adaptive immune responses. TLR5 is expressed on the surface of a range of host cells including epithelial cells, endothelial cells, macrophages, dendritic cells (DCs) and T cells^17–19^. As a member of the TLR family, TLR5 forms an important link between the innate and adaptive immune systems and plays a role in the maintenance of gut homeostasis. TLR5 interacts with the extracellular monomeric form of bacterial flagellin of both Gram-negative and Gram-positive bacteria, leading to activation of the NF-κB signalling pathway *via* the adaptor protein MyD88 and the serine kinase IRAK^20–22^. This can lead to systemic immune responses, stimulating the production of pro-inflammatory mediators including TNF-α, IL-1β, IL-6, IL-8, IL-12 and IL-23. A study by Cai *et al*. has shown that expression and activation of TLR5-associated pathways were elevated in breast carcinomas^23^. Furthermore, they demonstrated that flagellin activation of TLR5 in that context resulted in the local release of pro-inflammatory cytokines and anti-tumorigenic effects^23^.

Historically, flagellin has been studied as a virulence-associated trait but is also recognized as a host colonisation factor. Bacterial flagellin is characterised by highly conserved N- and C-terminal domains (D0 and D1 domains) which have been shown to interact directly with TLR5^21,24^. The hypervariable central region of flagellin (D2 and D3 domains) varies in size and structural organisation between species, and constitutes the main antigenic region of the protein^24,25^. Antigenic variation is thought to be one mechanism by which strains evolve to evade the host immune system^26^. Serologically distinct flagellins have been identified within bacterial species and have been used to track and type isolates^27,28^.

Herein, we characterised the immunostimulatory potential of *E. gallinarum* MRx0518, a human commensal bacterium with demonstrated anti-tumorigenic properties^12^. This is the first study to examine the role of *E. gallinarum* flagellin as potential immunogens in the human gut. This work provides insights into the molecular effectors through which strain MRx0518 elicits an immunostimulatory response in human intestinal epithelial cells (IECs), macrophages and DCs and potentially exerts its anti-tumorigenic activity *in vivo*.

## Results

### *Enterococcus gallinarum* MRx0518 induces a strong immunostimulatory response *in vitro*

**Figure 1:**
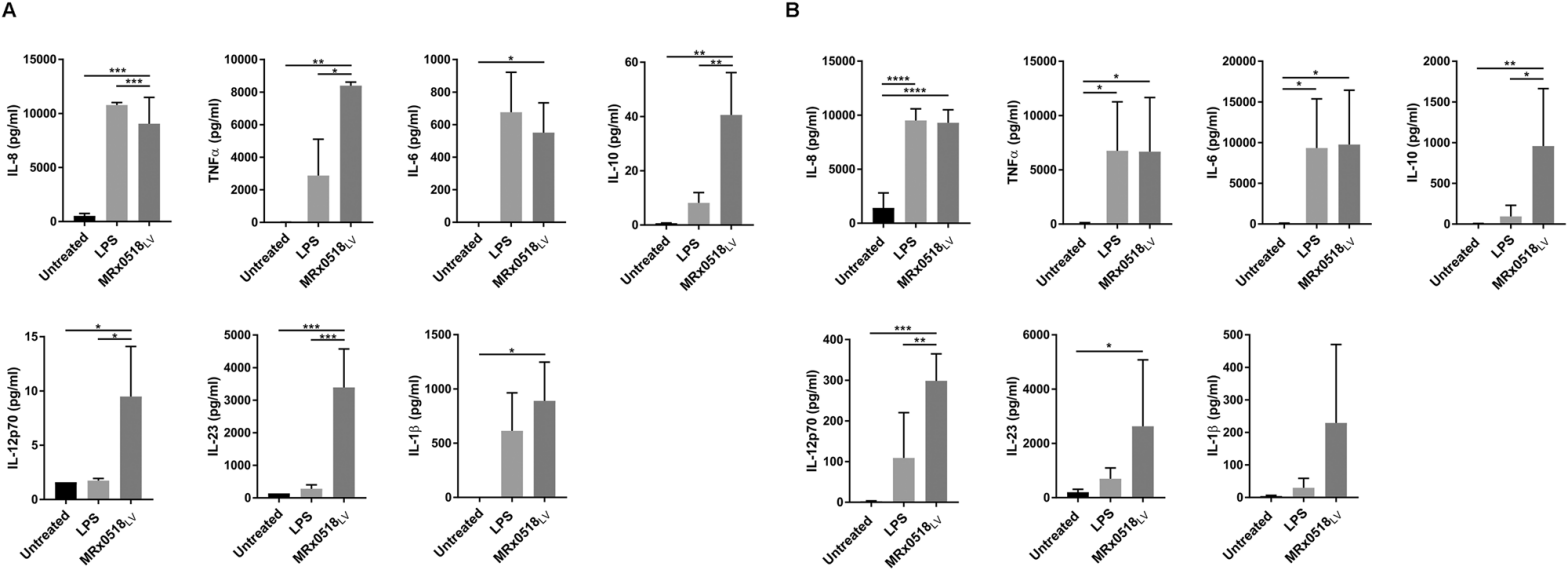
Cytokine production by THP-1-derived macrophages and monocyte-derived dendritic cells in response to *E. gallinarum* MRx0518. IL-8, TNFα, IL-6, IL-10, IL-12p70, IL-23, IL-1β concentrations (pg/ml) in (**A**) THP-1-derived macrophages and (**B**) monocyte-derived DCs cell-free supernatants after 1 h contact with *E. gallinarum* MRx0518 (MRx0518_LV_). A multiplicity of infection (MOI) of 10:1 was employed for both stimulation assays. A MOI of 1:1 was employed for IL-8 detection in macrophages, due to saturation of the assays at the MOI of 10:1. The effect of LPS on cytokines production is also shown as a positive control. The graphs represent an average of at least three biological replicates. Statistical comparisons were performed with GraphPad Prism (La Jolla, CA, USA) using ordinary one-way ANOVA analysis followed by Tukey’s multiple comparison tests. Statistically significant differences with the untreated control are shown on the graphs as *(p < 0.05), **(p < 0.01), ***(p < 0.001) and ****(p < 0.0001).

To perform an initial assessment of the immunostimulatory potential of *E. gallinarum* MRx0518, we measured the cytokine responses of two key innate immune cell types, THP-1-derived macrophages and monocyte-derived DCs, after stimulation with live MRx0518 cells (Fig. 1). We assessed a panel of pro- and anti-inflammatory cytokines involved in innate immunity and recruitment and activation of adaptive immune cells (IL-8, TNFα, IL-6, IL-10, IL-12p70, IL-23 and IL-1β). Both macrophages (Fig. 1A) and DCs (Fig. 1B) when unstimulated showed little to no production of the cytokines tested. As expected, a broadly consistent inflammatory profile was observed in both cell types in response to lipopolysaccharide (LPS), which was used as a pro-inflammatory response control (Fig. 1A,B). However, while LPS induced production of pro-inflammatory cytokines IL-6, IL-8 and TNFα in both macrophages and DCs, LPS-mediated expression of IL-12p70 and IL-1β was lower in DCs in comparison to macrophages (Fig. 1A,B). Compared to LPS, the cytokine production profiles were more consistent across the two cell types following *E. gallinarum* MRx0518 treatment (Fig. 1A,B). Both LPS and MRx0518 significantly induced IL-8 production in macrophages and DCs (*p* < 0.0001). MRx0518 also induced production of all other pro-inflammatory cytokines tested in both cell types. Significantly higher levels of TNFα (*p* < 0.001 and *p* < 0.01), IL-6 (*p* < 0.001 and *p* < 0.001) IL-12p70 (*p* < 0.05 and *p* < 0.001) and IL-23 (*p* < 0.001 and *p* < 0.05) in comparison to untreated cells were observed in macrophages and DCs. IL-1β production was also induced by MRx0518 stimulation in both cells types but only reached statistical significance in macrophages (*p* < 0.05). In addition to up-regulating the production of pro-inflammatory cytokines, MRx0518 treatment also induced a significant increase in IL-10 levels in comparison to LPS-treated and untreated cells. Of note, the variation observed in cytokine production by DCs (Fig. 1B) is most likely attributable to inherent donor heterogeneity. Overall, the data indicate that strain MRx0518 has a clear and potent immunostimulatory effect on host immune cells by inducing the production of a range of pro- and anti-inflammatory cytokines associated with both innate and adaptive immunity.

### *E. gallinarum* MRx0518 treatment affects gene expression in human intestinal epithelial cells

**Figure 2:**
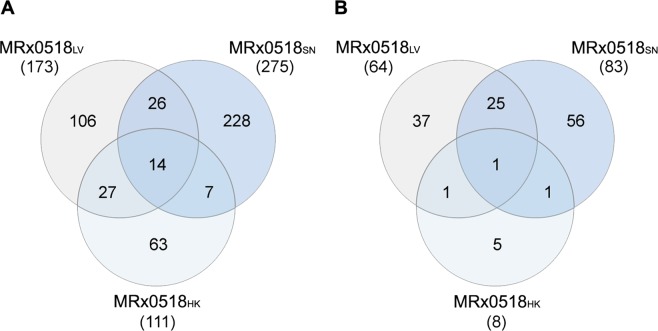
Transcriptomic analysis of the response of HT29-MTX cells to *E. gallinarum* MRx0518 treatments. Venn diagrams showing (**A**) up- and (**B**) down-regulated genes in HT29-MTX cells after 3 h contact with MRx0518 live (MRx0518_LV_), heat-killed (MRx0518_HK_) and culture supernatant (MRx0518_SN_) (MOI 100:1 or equivalent). Each treatment was compared to its respective control (cell culture media or YCFA). Diagrams were generated with InteractiVenn^62^.

IECs represent one of the primary points of contact between commensal bacteria and the host in the gut. A Transwell® co-culture system was employed to assess the transcriptional response of the mucin-secreting cell line HT29-MTX to treatment with MRx0518, using a Human Transcriptome Microarray (Fig. 2). Treatments with metabolically active cells (MRx0518_LV_), inactivated cells (MRx0518_HK_) or culture supernatants (MRx0518_SN_) were found to induce distinct host responses. Treatment with MRx0518_SN_ elicited the largest number of differentially expressed genes, inducing upregulation of 275 genes, 228 of which were not upregulated by other MRx0518 treatments (Supplementary Table S1). MRx0518_LV_ and MRx0518_HK_ induced the upregulation of 106 and 63 genes respectively that were not upregulated in MRx0518_SN_-treated cells. Similarly, the MRx0518_SN_ also induced the downregulation of the largest number of genes in IECs (Fig. 2). Only 14 upregulated genes and one downregulated gene were common to all treatment groups (Supplementary Table S1). MRx0518_HK_ cells had the least impact on IEC transcription levels (Fig. 2). Despite MRx0518_SN_ treatment inducing the largest number of differentially expressed genes in IECs, pathway enrichment analysis of the transcriptomic data indicated that MRx0518_LV_ had the largest impact on physiological pathways, with over-representation of pathways involved in innate inflammatory responses, interferon signalling and apoptosis (Supplementary Fig. S1). Of particular interest was the upregulation of *CCL20* (~20-fold) and *CXCL8* (~5-fold) in MRx0518_LV_- and MRx0518_SN_-treated cells, both of which play a role in immune cell recruitment (Table 1). *NFKBIA* and *TNFAIP3*, genes involved in regulating NF-κB signalling, were also significantly upregulated in MRx0518_LV_-treated cells. *ICAM1* was significantly upregulated in MRx0518_LV_-treated cells, but not in MRx0518_SN_-treated cells (Table 1). HT29-MTX cells demonstrated modest upregulation of *CXCL1* expression (2.41-fold) in response to MRx0518_LV_, which was not observed with other treatments.

**Table 1:** Immunomodulatory genes selected for further analysis. Genes were filtered based on a fold change ≥ 1.5, *p* < 0.05, coding transcripts only and the presence of a gene symbol. n.s.: not significant.

Gene	Description	MRx0518_LV_		MRx0518_HK_		MRx0518_SN_	
		Fold change	*p*-value	Fold change	*p*-value	Fold change	*p*-value
*CCL20*	Chemokine (C-C motif) ligand 20	20.59	1.10e-13	n.s.	n.s.	8.22	2.06e-11
*CXCL8*	Chemokine (C-X-C motif) ligand 8	4.87	5.24e-13	n.s.	n.s.	1.56	3.03e-05
*ICAM1*	Intercellular adhesion molecule 1	4.03	2.03e-09	n.s.	n.s.	n.s.	n.s.
*NFKBIA*	NF-κB inhibitor alpha	3.99	5.51e-08	n.s.	n.s.	1.61	0.0015
*TNFAIP3*	TNF alpha-induced protein 3	2.95	3.58e-10	n.s.	n.s	n.s	n.s
*CXCL1*	Chemokine (C-X-C motif) ligand 1	2.41	1.13e-08	n.s.	n.s	n.s.	n.s

### A protein in MRx0518 culture supernatant activates NF-κB and TLR5 reporter cells

**Figure 3:**
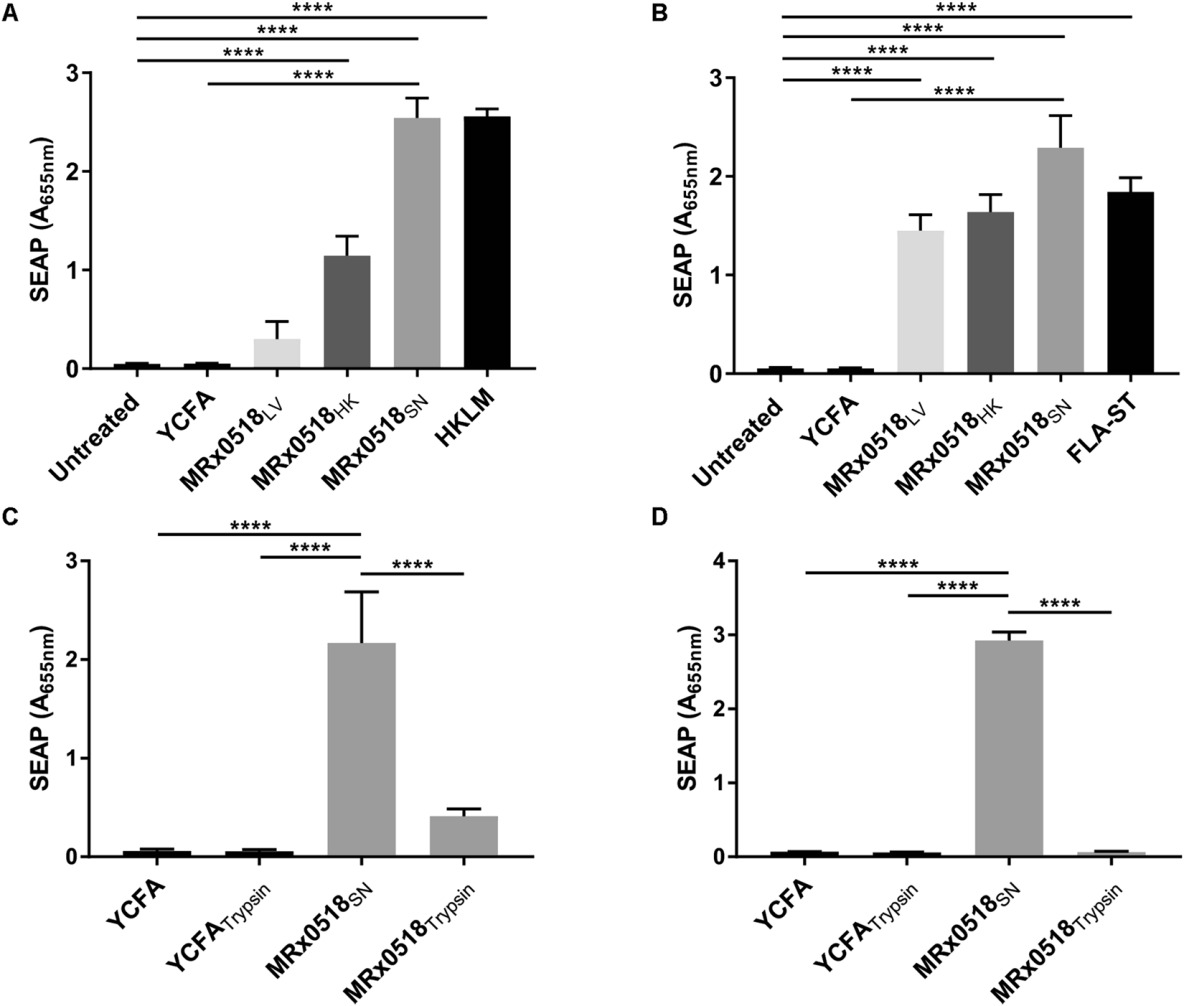
Activation of NF-κB and TLR5 pathway by *E. gallinarum* MRx0518 treatments. NF-κB (**A**) and TLR5 (**B**) activation after 22 h incubation with *E. gallinarum* MRx0518 (MRx0518_LV_), heat-killed MRx0518 (MRx0518_HK_) and culture supernatant (MRx0518_SN_) in HEK-Blue™ hTLR5 and THP1-Blue™ NF-kB reporter cell lines. A MOI of 10:1 was used with MRx0518_LV_ and a 100:1 MOI equivalent was used with MRx0518_HK_ and MRx0518_SN._ Heat-killed *Listeria monocytogenes* (HKLM) and *Salmonella* Typhimurium flagellin (FLA-ST) were used as positive controls for each cell line and YCFA was included as a negative control for MRx0518_SN_. NF-κB **(C)** and TLR5 **(D)** activation after 22 h incubation with *E. gallinarum* MRx0518 culture supernatant (MRx0518_SN_) and trypsin-treated supernatant (MRx0518_Trypsin_) (MOI 100:1 equivalent). Each graph represents an average of at least three biological replicates. Statistical comparisons were performed with GraphPad Prism using ordinary one-way ANOVA analysis followed by Tukey’s (**A**,**B**), Dunnett’s (**C**) or Sidak’s (**D**) multiple comparison tests. Statistically significant differences with the relevant control are shown on the graphs as ****(*p* < 0.0001).

The immunostimulatory response observed in macrophages, DCs and IECs following stimulation with MRx0518 appears to be exerted, in part, through NF-κB signalling. In order to confirm activation of this signalling pathway, we examined the effect of MRx0518 treatments (MRx0518_LV_, MRx0518_HK_ and MRx0518_SN_) on NF-κB and TLR5 reporter cell lines (Fig. 3). NF-κB activation was assessed by measuring the expression of the secreted embryonic alkaline phosphatase (SEAP) reporter gene. MRx0518_LV_ did not activate NF-κB reporter cells but activated TLR5 reporter cells (*p* < 0.0001) (Fig. 3A,B). The lack of SEAP detection in NF-κB reporter cells is likely due to the growth of MRx0518 in the cell culture media during the 22 h incubation which possibly impacted the viability of the reporter cells rather than genuine absence of signalling activation. MRx0518_HK_ induced a strong response in the TLR5 reporter cells, which was slightly higher than that observed in the NF-κB reporter cells (*p* < 0.0001 for both cell lines in comparison to untreated cells) (Fig. 3A,B). Both reporter cell lines were activated by MRx0518_SN_, to the same extent as their respective positive controls (Fig. 3A,B). Overall, MRx0518_SN_ was the most potent stimulant of both NF-κB and TLR5. These results, combined with the transcriptional response of HT29-MTX cells to MRx0518_SN_, prompted us to investigate the active component in this fraction. In an effort to identify the nature of molecules responsible for the observed host response, we treated MRx0518_SN_ with a range of enzymes (*i.e*. DNase, proteases and apyrase). Trypsin treatment had the greatest effect on activation of NF-κB and TLR5 reporter cells, while other enzymatic treatments had smaller effects (data not shown). TLR5 activation was completely abolished by trypsin treatment, whereas low but detectable activation remained in the NF-κB reporter cells (*p* < 0.0001 when compared to MRx0518_SN_) (Fig. 3C,D). These assays established that a molecule of proteinaceous nature was present in MRx0518_SN_ which was most likely responsible for TLR5-mediated NF-κB activation. However, residual NF-κB activation (Fig. 3C) also suggested that other molecules in MRx0518_SN_ that are not affected by trypsin digestion may contribute to NF-κB signalling. Flagellin is the only known TLR5 ligand, and both expression profiling and phenotypic observations indicated that MRx0518 expresses flagellin in its late log growth phase and is motile under *in vitro* conditions (Supplementary Fig. S2). NanoLC-MS/MS analysis confirmed the presence of flagellin in high abundance in MRx0518_SN_ (Supplementary Table S2), strongly suggesting that flagellin was the molecule responsible for the observed NF-κB activation in the reporter assays.

### Flagellin is responsible for the activation of NF-κB and TLR5 reporter cells

**Figure 4:**
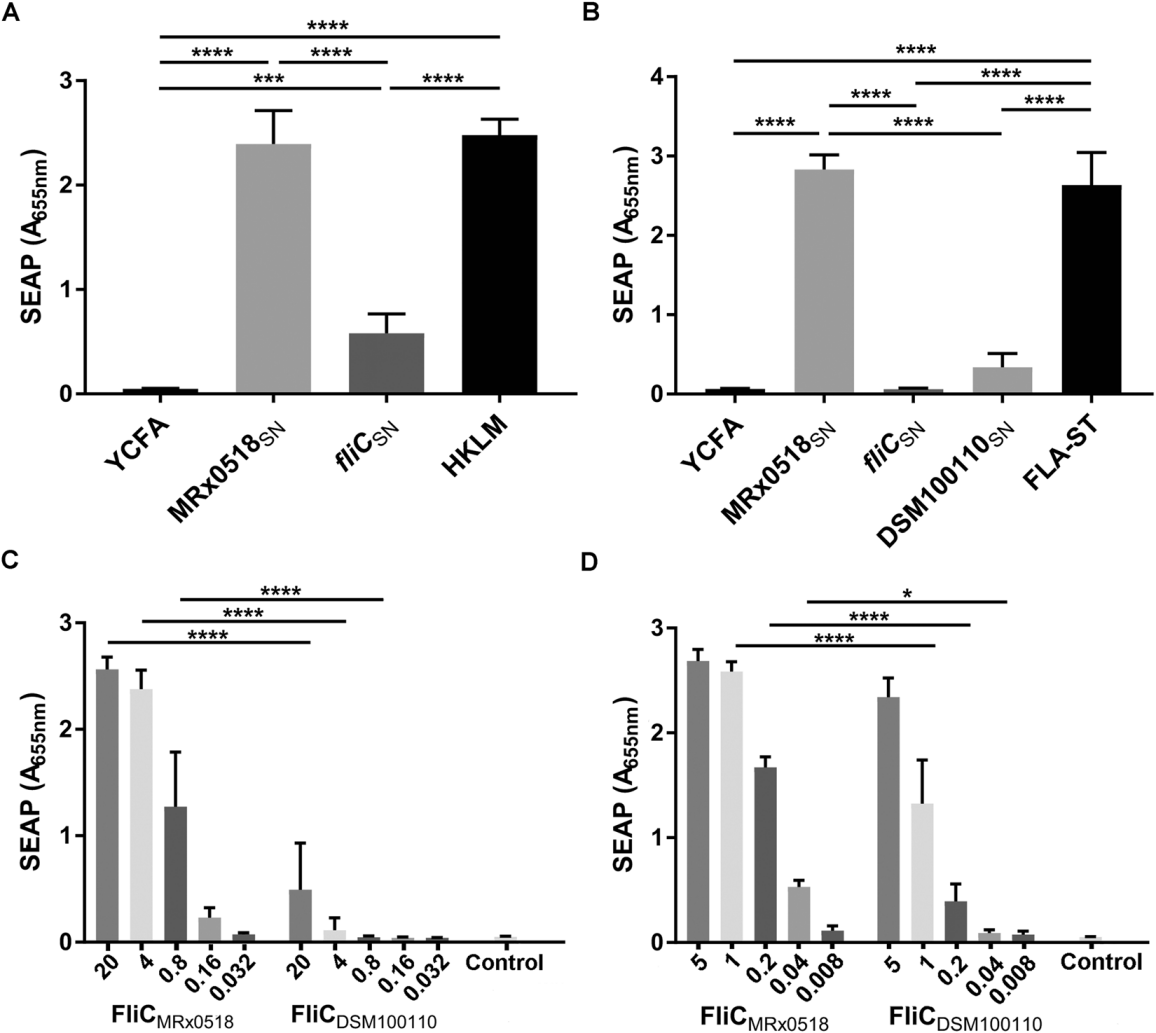
Flagellin plays a role in *E. gallinarum* MRx0518 immunostimulatory effect. NF-κB (**A**) and TLR5 (**B**) reporter assays with MRx0518 (MRx0518_SN_), MRx0518 *fliC*::pORI19 (*fliC*_SN_) and DSM100110 (DSM100110_SN_) culture supernatants (MOI 100:1 equivalent). NF-κB (**C**) and TLR5 (**D**) reporter assays with a range of concentrations of *E. gallinarum* MRx0518 and DSM100110 purified recombinant flagellins (FliC_MRx0518_ and FliC_DSM100110_). The ‘Control’ bar corresponds to the empty vector control. Each graph represents an average of at least three biological replicates. Reporter cells were incubated with treatments for 22 h. Statistical comparisons were performed with GraphPad Prism using ordinary one-way ANOVA analysis followed by Tukey’s (**A**,**B**) or Sidak’s (**C**,**D**) multiple comparison tests. Statistically significant differences with the relevant control are shown on the graphs as *(*p* < 0.05), ***(*p* < 0.001) and ****(*p* < 0.0001).

An insertion mutation in the flagellin gene (*fliC*) in *E. gallinarum* MRx0518 (strain MRx0518 *fliC*::pORI19) was generated. Genetic manipulations in *E. gallinarum* had not been described in the literature prior to this study, it was therefore necessary to develop a transformation protocol for strain MRx0518. Electrocompetent cells were successfully generated by growing bacterial cultures in sub-inhibitory concentrations of glycine^29^ followed by mutanolysin and lysozyme treatments to further weaken the cell wall peptidoglycan layer (see Material and Methods for details). The *fliC* gene was disrupted by homology-driven insertion of the suicide plasmid pORI19^30^. Insertion of pORI19 within the *fliC* gene was confirmed by DNA sequencing and the non-motile phenotype of the resulting mutant strain was confirmed *in vitro* (Supplementary Fig. S2). Both MRx0518_SN_ and MRx0518 *fliC*::pORI19 culture supernatant (*fliC*_SN_) were tested in the NF-κB and TLR5 reporter assays along with culture supernatant from an additional *E. gallinarum* strain, DSM100110 (DSM100110_SN_) (Fig. 4A,B). Strain DSM100110 is a murine isolate which was chosen for its highly-motile phenotype *in vitro* (Supplementary Fig. S2). A significant reduction of NF-κB activation (approximately 75% compared to MRx0518_SN_) was observed for *fliC*_SN_-treated NF-κB reporter cells (*p* < 0.0001) (Fig. 4A). The presence of additional stimulatory molecules in *fliC*_SN_ may have contributed to the observed residual activation of NF-κB signalling, as previously noted for trypsinized-MRx0518_SN_ (Fig. 3C). Inactivation of the flagellin gene completely abolished TLR5 activation (no observable difference with the YCFA culture medium control) and was significantly reduced in comparison to MRx0518_SN_ (*p* < 0.0001) (Fig. 4B). Interestingly DSM100110_SN_ induced very little activation of the TLR5 reporter cells (not statistically significant when compared to YCFA). Analysis by nanoLC-MS/MS of the protein content of DSM100110_SN_ revealed the absence of flagellin (data not shown), which explains the observed lack of TLR5 activation elicited by this strain (Fig. 4B).

The lack of flagellin in DSM100110_SN_ limited our ability to determine the immunogenic potential of its flagellin in supernatant-stimulation assays. Flagellins from strains MRx0518 and DSM100110 were therefore overexpressed and purified as N-terminally his-tagged recombinant proteins (Supplementary Fig. S3) and used to perform a dose-response assay. Both recombinant flagellins were capable of activating NF-κB and TLR5 reporter cells (Fig. 4C,D). Lower protein concentrations stimulated a strong response in TLR5 reporter cells in comparison to the NF-κB reporter cells. This was perhaps unsurprising as flagellin is known to interact directly with TLR5^20^ and this TLR5 reporter cell line is expected to be 20–100 fold more responsive than cell lines that express basal levels of TLR5, as indicated by the manufacturer. Purified FliC_MRx0518_ showed comparable levels of activation to MRx0518_SN_ in both reporter cell lines, with saturation levels dropping at concentrations less than 1 ng/ml. FliC_DSM100110_ was weakly active in the NF-κB reporter assay at the concentrations tested (Fig. 4C). At concentrations greater than 5 ng/ml, both recombinant proteins induced comparable responses in TLR5 reporter cells, which were not significantly different (Fig. 4D). However, at lower concentrations FliC_MRx0518_ was significantly more stimulatory than FliC_DSM100110_ at equivalent concentrations (*p* < 0.0001 for tests at 0.2 and 1 ng/ml and *p* < 0.05 for 0.04 ng/ml). The same trend was observed in the NF-κB reporter cells (*p* < 0.0001 for 0.8, 4 and 20 ng/ml).

### FliC_MRx0518_ and FliC_DSM100110_ display sequence divergence and reside in distinct clusters of a FliC phylogenetic tree

**Figure 5:**
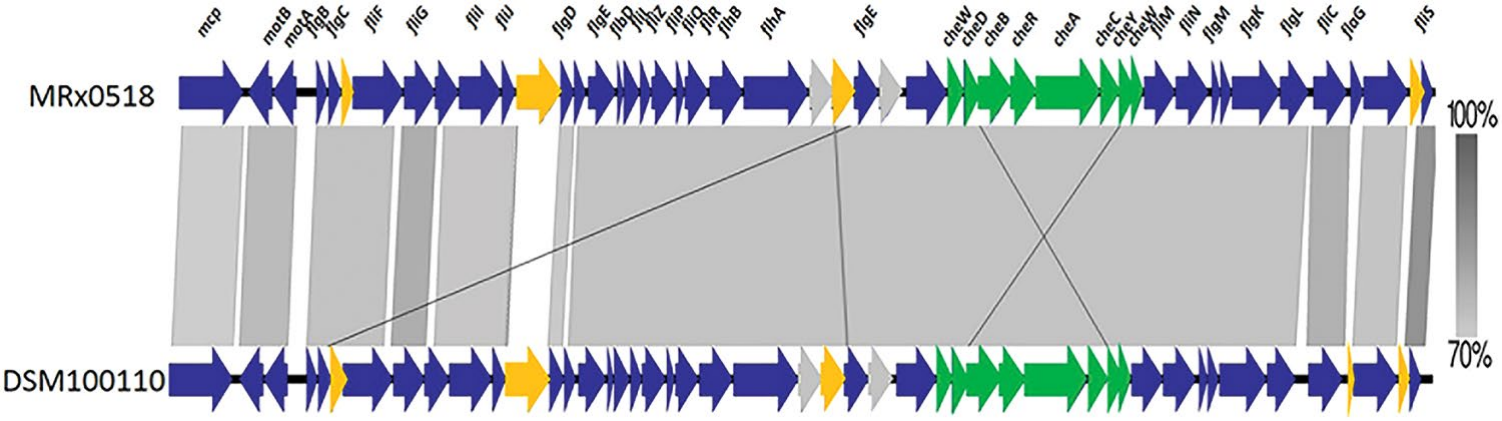
Sequence alignment of the flagellar loci of *E. gallinarum* MRx0518 and *E. gallinarum* DSM100110. A linear comparison of the BLASTN matches between the flagellar loci of of *E. gallinarum* strains MRx0518 and DSM100110. Vertical grey-coloured blocks between sequences indicate regions of shared nucleotide ID. The gradient of the grey colour corresponds to the percentage of shared nt ID. The genes in each element are coloured according to their function, as follows: blue (biosynthesis), green (chemotaxis), grey (other function) and yellow (hypothetical proteins).

FliC_MRx0518_ displayed a higher capacity to stimulate both TLR5 and NF-κB than FliC_DSM100110_ at low concentrations. This prompted the further examination of the flagellar loci and particularly the FliC protein sequences of both strains. Organisationally-conserved 40-kb motility loci were identified in the genome sequences of strains MRx0518 and DSM100110, both of which encode 47 contiguous genes (Fig. 5). Gene organisation was similar to that of other motile enterococci^4^ and both strains share 69.3% nucleotide (nt) identity (ID) over the length of the operon. Indels are present between the two loci, which result in changes to the start and stop sites of a number of homologous genes but are not predicted to result in the formation of pseudogenes. Each strain encodes a single FliC protein which are 360 amino acid (aa) and 361 aa long in MRx0518 and DSM100110 respectively and share 77.2% aa identity (Fig. S4 and Supplementary Dataset S1). Modelling of the structure of FliC_MRx0518_ revealed the presence of three domains (Fig. S4), as predicted using the Phyre^2^ server^31^ (data not shown).

**Figure 6:**
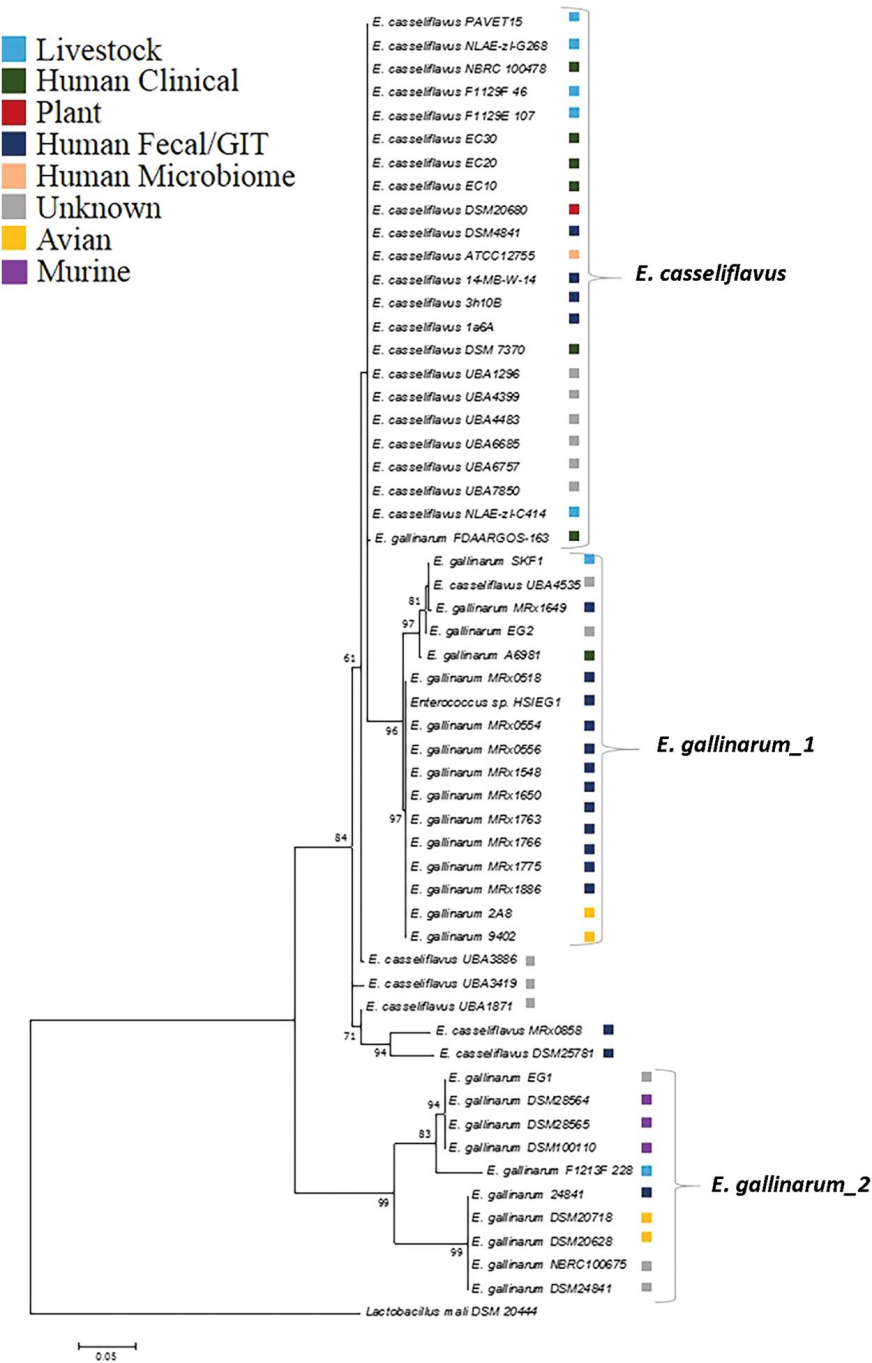
Phylogenetic analysis of the FliC protein of *E. gallinarum* and *E. casseliflavus*. The FliC sequences of selected strains (Supplementary Table S3) were aligned with MUSCLE^59^. The evolutionary history was inferred by using the maximum likelihood method based on the Le_Gascuel_2008 model^61^, using MEGA7 software^60^. Statistical support (above 60%) was estimated with bootstraps and is indicated at branch nodes. Well-defined clades are indicated. The type strain *Lactobacillus mali* DSM20444 was used as an outgroup for analyses. The origins of strains are indicated where the information was available on the National Center for Biotechnology Information (NCBI) website (https://www.ncbi.nlm.nih.gov).

Phylogenetic analyses indicated that the FliC proteins of *E. gallinarum* and *E. casseliflavus* branch closely to the FliC proteins of motile lactobacilli (Supplementary Fig. S5, Supplementary Dataset S1)^32,33^. In order to assess the level of diversity within the flagellin of these closely related species, the FliC sequences of 15 *E. gallinarum* and 3 *E. casseliflavus* strains derived from the 4D Pharma plc and DSMZ culture collections (Supplementary Table S3), together with those available in public databases (11 *E. gallinarum* and 27 *E. casseliflavus*), were assessed by comparative analyses. The FliC proteins of *E. gallinarum* varied in length from 352 aa to 361 aa, while the *E. casseliflavus* FliC proteins varied between 357 aa and 361 aa. Between 75.82% and 100% aa ID was observed among the examined proteins, with several *E. gallinarum* FliC proteins displaying higher levels of sequence homology to *E. casseliflavus* FliC, than to each other (Supplementary Fig. S4, Supplementary Dataset S1). The highest level of sequence divergence both within and between the *E. gallinarum* and *E. casseliflavus* FliC proteins was observed in the D2 region whereas the D0 and D1 regions were more highly conserved (Fig. S4). The regions known to be critical for TLR5 interaction in other bacterial species^21,34^ were found to be conserved (residues 87–96) in all strains examined. Three distinct clusters were present within the FliC-based Maximum Likelihood phylogenetic tree shown in Fig. 6, with two well-supported *E. gallinarum* clusters evident (*E. gallinarum*_1 and *E. gallinarum*_2) and the majority of *E. casseliflavus* strains grouping together. Interestingly, strains MRx0518 and DSM100110 were resident in distinct clusters, each of which broadly represent their sources of isolation (Fig. 6).

### Inactivation of the flagellin gene abolishes MRx0518 immunogenic effects in IECs

**Figure 7:**
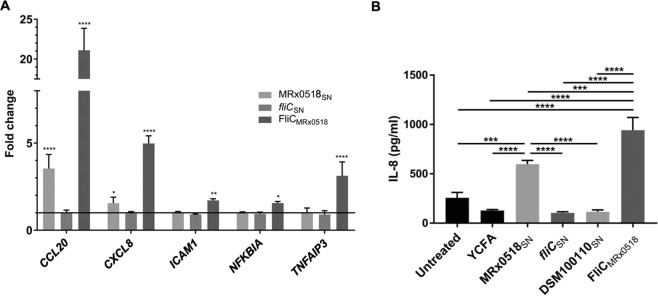
Inactivation of the flagellin gene in *E. gallinarum* MRx0518 abolishes its immunostimulatory profile. (**A**) Changes in gene expression in HT29-MTX after 24 h co-culture with MRx0518 and MRx0518 *fliC*::pORI19 culture supernatants (MRx0518_SN_ and *fliC*_SN_) (MOI 100:1 equivalent) and 1 µg/ml purified MRx0518 recombinant flagellin (FliC_MRx0518_), measured by qPCR (fold change compared to the YCFA or the empty vector control, as appropriate). Statistical comparisons were performed with GraphPad Prism on ΔCT values using two-way ANOVA followed by Tukey’s multiple comparison tests. Statistically significant differences in comparison to the YCFA or empty vector control group as appropriate are shown on the graph as *(*p* < 0.05), **(*p* < 0.01) and ****(*p* < 0.0001). (**B**) IL-8 concentrations (pg/ml) detected by ELISA assay in HT29-MTX cell-free supernatant after 24 h co-culture with MRx0518 (MRx0518_SN_), MRx0518 *fliC*::pORI19 (*fliC*_SN_) and DSM100110 (DSM100110_SN_) culture supernatants (MOI 100:1 equivalent), and 1 µg/ml purified MRx0518 recombinant flagellin (FliC_MRx0518_). YCFA was included as a negative control. Statistical comparisons were performed with GraphPad Prism using an ordinary one-way ANOVA followed by Tukey’s multiple comparison test. Statistically significant differences with the relevant control are shown on the graphs as ***(*p* < 0.001) and ****(*p* < 0.0001).

In order to confirm the involvement of flagellin in the observed immunostimulatory effects of strain MRx0518, the impact of MRx0518_SN_ and *fliC*_SN_ together with FliC_MRx0518_ recombinant flagellin on gene expression and cytokine production levels in IECs were tested. The changes in IEC gene expression following stimulation with MRx0518_SN_, *fliC*_SN_ and FliC_MRx0518_ were investigated using a targeted panel of qPCR primers, designed based on the transcriptional profiles of IECs in response to the MRx0518 treatments described above (Table 1, Fig. 7A). The expression of *NFKBIA* was unchanged, despite a slight upregulation being observed in the microarray-derived data (1.61-fold). MRx0518_SN_ significantly induced the expression of the *CCL20* and *CXCL8* genes (*p* < 0.0001 and *p* < 0.05 respectively compared to the YCFA treatment) (Fig. 7A), which was consistent with the upregulation previously observed. *fliC*_SN_ had no effect on expression levels of the five genes tested. Co-culture of HT29-MTX cells with *fliC*_SN_ did not induce the stimulatory response observed with MRx0518_SN_ which strongly supports the role of flagellin as a major effector of MRx0518 immunogenicity. This was further confirmed by co-culturing HT29-MTX cells with recombinant MRx0518 flagellin. The addition of FliC_MRx0518_ led to a significant (*p* < 0.05) upregulation in the expression of all five genes in the panel, with fold changes higher than those observed with MRx0518_SN_ (Fig. 7A). The levels of IL-8 secreted by HT29-MTX cells stimulated with MRx0518_SN_, *fliC*_SN_, DSM100110_SN_ and FliC_MRx0518_ was measured in cell-free supernatants following 24 h co-culture (Fig. 7B). MRx0518_SN_ induced a significant release of IL-8 in IECs in comparison to cells treated with YCFA (*p* < 0.0001). The inactivation of the flagellin gene in the MRx0518 strain reduced IL-8 secretion to levels comparable to those observed with YCFA. Treatment of cells with recombinant flagellin strongly stimulated IL-8 secretion in comparison to the untreated and YCFA groups (*p* < 0.0001) but also in comparison to cells treated with MRx0518_SN_ (*p* < 0.001). In contrast, DSM100110_SN_ had no observable impact on IL-8 stimulation (Fig. 7B).

## Discussion

*Enterococcus gallinarum* MRx0518 is a candidate live biotherapeutic, isolated from a healthy human faecal sample. Oral delivery of this strain has demonstrated anti-tumour efficacy in murine models of breast, lung and renal carcinomas^12^. Only a limited number of studies have characterised strains of this species in any detail, and fewer still that have examined the interactions of *E. gallinarum* strains with the immune system^35,36^. To begin to understand how strain MRx0518 interacts with the host, we examined its effect upon IECs, macrophages and DCs, host cells which have distinct roles in the innate immune response. DCs are capable of priming T cells at distal sites and stimulating a homing response to drive T cell accumulation to sites of inflammation. Macrophages tend to act locally to maintain homeostasis and can induce secondary activation of T cells. Both cell types come into direct contact with luminal bacteria in the gut but can also play a role in anti-tumour immunity at distal tumour sites. MRx0518_LV_ elicited a strong and consistent pro-inflammatory signature in both macrophages and DCs, at levels similar to or higher than those elicited by the control inflammatory stimulant LPS. MRx0518_LV_ significantly elevated levels of TNFα, a known regulator of IL-6 and IL-8 production, in both DCs and macrophages. Similarly, a study by van den Bogert and colleagues found that *E. gallinarum* HSIEG1 is also capable of inducing cytokine secretion *in vitro*^35^. IL-10, a cytokine well-described for its anti-inflammatory and tolerogenic effects, was also induced by MRx0518_LV_ treatment and can actively suppress the expression of IL-6, IL-12, IL-1β and TNFα. Given the elevation of both pro- and anti-inflammatory cytokines in response to MRx0518 treatment, it is noteworthy that IL-10 and IL-6 are reciprocal cytokines, both utilising the activity of transcription factor STAT3 to alter cellular responses with broadly opposing effects^37^. IL-10 is not a pan-inhibitory cytokine of inflammatory responses; it is known to activate and increase CD8α cytotoxic capacity which may be significant for anti-tumour responses^38,39^.

High levels of IL-8 production were observed in macrophages and DCs in response to MRx0518_LV_ exposure. Additionally, MRx0518_LV_, MRx0518_SN_ and purified flagellin all induced expression of *CCL20* and *CXCL8* in HT29-MTX cells; the products of which are implicated in the recruitment of immune cells and the subsequent activation of the adaptive branch of the immune system. Similarly, *Salmonella*-derived flagellin has been shown to stimulate expression of the *CCL20* gene in Caco-2 cells^40^ and *E. coli* flagellin has been shown to induce secretion of IL-8 and CCL20 in HT29–19A and Caco-2 cells^41^.

Flagellin is a potent immunostimulant which acts through TLR5 and has been exploited in recent years for its capacity as a vaccine adjuvant and its anti-tumorigenic efficacy^13^. Purified MRx0518 flagellin showed significant activation of TLR5 in reporter cells and proved more potent than DSM100110 flagellin at equivalent nanomolar concentrations. Given the body of work that is emerging regarding the role of TLRs and their associated ligands in anti-cancer therapies, the potential contribution of flagellin to MRx0518 anti-tumour activity warrants further investigation. The administration of *S*. Typhimurium flagellin has been shown to reduce tumour growth and cell proliferation in colon and breast cancer cells^23,42^. An elegant study by Cai *et al*. demonstrated that 80% of breast carcinoma tissues tested were found to be positive for TLR5 expression and that TLR5-signalling was also upregulated in breast carcinomas^23^. They concluded that flagellin-mediated TLR5 activation is involved in modulation of the tumour microenvironment and mediates its anti-tumorigenic effect through pro-inflammatory cytokine induction. A flagellin from *V. vulnificus* expressed in an attenuated strain of *S*. Typhimurium was shown to be effective in tumour growth reduction in several murine cancer models when delivered intravenously^14^. Interestingly, Zheng *et al*. showed that *Salmonella* and flagellin demonstrate complementarity to recruit and activate immune cells, through colonization of the tumour site and interaction with TLR5 respectively^14^.

Activation of TLRs on the surface of tumours may require transport or delivery of flagellin to distal tumour sites which could be achieved through translocation of the bacteria or their components from the gut. Manfredo-Viera *et al*. recently demonstrated that *E. gallinarum* was able to translocate from the murine gut to induce an autoimmune response in immunocompromised mice^36^. Translocation of another enterococcal species *E. hirae* to secondary lymphoid organs has been shown to enhance efficacy of a chemotherapeutic agent^9^. Additionally, *E. gallinarum* was found to be overly abundant in patients who responded to treatment with anti-PD1^11^, suggesting a potential role for this species in patient responsiveness to ICI treatments. Studies are currently ongoing to investigate the ability of MRx0518 to translocate from the gut to extra-intestinal sites. Testing the translocation potential of MRx0518-derivatives, including flagellin, is also underway using FliC_MRx0518_-directed antibodies.

Inter- and intra-species comparative analysis of the FliC proteins of *E. gallinarum* and *E. casseliflavus* indicate that the D0 and D1 domains are highly conserved while the majority of variability lies within the D2 domains, as observed for flagellin of other species^25^. The sequence divergence displayed in the FliC sequence in *E. gallinarum* is comparable with that of *C. difficile*^43^ and *E. coli*^44^ and is less than that reported for *P. aeruginosa*^45^ and *B. thuringiensis*^46^. Antigenic variation in the FliC sequence may contribute to differences in immunogenic potential of *E. gallinarum* strains. However, it is yet to be determined to what extent the variance observed in the D2 domains of FliC_MRx0518_ and FliC_DSM100110_ contributes to the immunogenic profiles of these strains.

Inactivation of flagellin in MRx0518 resulted in complete abrogation of TLR5-mediated activation of NF-κB. However, some residual activity remained in the NF-κB reporter cells when treated with *fliC*_SN_. This suggests the involvement of additional or complementary bacterial effectors present in culture supernatants. Of particular interest was the identification of enolase as the most abundant protein in the MRx0518_SN_ (Supplementary Table S2) as enolase has been shown to play a role in host-interactions in lactic acid bacteria, through plasminogen binding^47^. Small molecules such as ATP and CpG DNA, can act synergistically with flagellin to trigger host immune responses^48–50^. The potential contribution of these molecules to the observed immunogenic effects of MRx0518 warrants further investigation.

Taken together, these data demonstrate that *E. gallinarum* MRx0518, and more specifically its flagellin, is a strong immunostimulant of both immune and intestinal epithelial cells. Importantly FliC_MRx0518_ displays higher potency than FliC_DSM100110_. The extent of the activity of MRx0518 flagellin *in vivo* remains to be determined. In this context, MRx0518 derivatives are currently being investigated in murine cancer models in order to shed light on their influence on the previously established therapeutic effect of *E. gallinarum* MRx0518.

## Material and Methods

### Bacterial strains, plasmids and culture conditions

*E. gallinarum* strains were routinely cultured in Yeast extract, Casitone, Fatty Acid media (YCFA, E&O Laboratories, Bonnybridge, Scotland, UK) at 37 °C in an anaerobic cabinet (Don Whitley Scientific, Shipley, England, UK). Late log phase cultures were grown for approximately 3 h (10% inoculum). *E. coli* strains were grown in Luria-Bertani broth at 20 °C or 37 °C in aerobic conditions with shaking (180–200 rpm). Growth media were supplemented with erythromycin (20 μg/ml for *E. gallinarum* and 100 μg/ml for *E. coli*), ampicillin (100 μg/ml) and kanamycin (25–50 μg/ml) (Sigma-Aldrich, Gillingham, England, UK), where appropriate. Bacterial strains and plasmids used in this study are listed in Supplementary Table S3.

### Preparation of bacterial fractions for co-culture assays

Late log phase bacterial cultures were centrifuged at 5000 x g for 5 min at room temperature to generate bacterial fractions. Pelleted cells were washed once in phosphate-buffered saline (PBS) (Sigma-Aldrich) and resuspended in antibiotic-free cell culture media to the appropriate dilution (live fraction, MRx0518_LV_). Culture supernatants were harvested and filtered through a 0.22 μm pore size filter and diluted in water to provide equivalents for the live fraction described above (supernatant fraction, MRx0518_SN_). Bacterial cultures were heat-inactivated for 40 min at 80 °C and prepared as described above for the live fraction (heat-killed fraction, MRx0518_HK_). Viable cell counts were determined by spread plating. When required, culture supernatants were digested with 500 μg/ml trypsin or an equivalent volume of Hank’s balanced salt solution (HBSS) (Thermo Fisher Scientific, Waltham, MA, USA) as a mock digestion control for 1 h at 37 °C, followed by inactivation with 10% (v/v) foetal bovine serum (FBS) (Sigma-Aldrich).

### Immortalised cell lines and growth conditions

THP-1 cells (Public Health England, Salisbury, England, UK) were routinely grown in RPMI 1640 supplemented with 10% (v/v) FBS, 2 mM L-glutamine, 100 U/ml penicillin, 100 μg/ml streptomycin (cRPMI). HT29-MTX-E12 cells (Public Health England) were routinely cultured in Dulbecco’s Minimal Eagle’s Medium (DMEM) supplemented with 10% (v/v) FBS, 4 mM L-glutamine, 4.5 mg/ml glucose, 8.9 μg/ml L-alanine, 15 μg/ml L-asparagine, 13.3 μg/ml L-aspartic acid, 14.7 μg/ml L-glutamic acid, 7.5 μg/ml glycine, 11.5 μg/ml L-proline, 10.5 μg/ml L-serine, 100 U/ml penicillin, 100 μg/ml streptomycin and 0.25 μg/ml amphotericin B (cDMEM). Cells were seeded into assay vessels and cultured for nine days, following which they were washed twice with HBBS and placed into co-culture medium (cDMEM without antibiotic and supplemented with 5 μg/ml apo-transferrin and 0.2 μg/ml sodium selenite). HEK-Blue™-hTLR5 cells (InvivoGen, San Diego, CA, USA) were grown in DMEM supplemented with 10% (v/v) FBS, 4 mM L-glutamine, 4.5 mg/ml glucose, 100 U/ml penicillin, 100 μg/ml streptomycin, 100 μg/ml Normocin™ (InvivoGen), 30 μg/ml blastocydin and 100 μg/ml xeocin to 90% density. THP1-Blue™ NF-kB cells (InvivoGen) were grown in RPMI 1640 supplemented with 10% (v/v) FBS, 2 mM L-glutamine, 100 U/ml penicillin, 100 μg/ml streptomycin, 25 mM HEPES, 100 μg/ml Normocin™, 10 μg/ml blastocydin. All reagents were supplied by Sigma-Aldrich unless otherwise specified. Immortalised cell lines were routinely grown at 37 °C in 5% CO_2_ atmosphere.

### Immortalised and primary cells stimulation

THP-1 cells were differentiated into macrophages by the addition of 5 ng/ml phorbol 12-myristate 13-acetate (PMA) (Sigma-Aldrich) to the culture media for 48 h. Cells were plated in 96-well plates (200,000 cells/well) in cRPMI without PMA, antibiotics and FBS and incubated for 3 h. Treatments (live bacteria at a MOI of 10:1 or a MOI of 1:1 for IL-8 detection as saturation was obtained with the 10:1 MOI) and controls (50 ng/ml LPS or PBS) were then added and incubated for 1 h at 37 °C under anaerobic conditions. Culture medium was then replaced with cRPMI and incubated for 24 h under standard growth conditions. Cell-free supernatants were then harvested, centrifuged for 3 min at 10,000 x g at 4 °C and stored at −80 °C for cytokine detection. Human PBMCs, obtained from STEMCELL Technologies (Vancouver, Canada) from healthy donors, were used to isolate primary monocyte populations by negative selection using a Human Monocyte Isolation kit. Monocytes were then differentiated into immature dendritic cells by incubation with 20 ng/ml recombinant human IL-4 and 50 ng/ml recombinant human GM-CSF for 8 days at 37 °C in a 5% CO_2_ atmosphere in cRPMI supplemented with 55 μM 2-mercaptoethanol. Immature dendritic cells were recovered, washed, resuspended in cRPMI medium without antibiotics and plated in 96-well plates (200,000 cells/well). Treatments (live bacteria at a MOI of 10:1) and controls (100 ng/ml LPS or cRPMI) were added to the cells and incubated for 1 h at 37 °C under anaerobic conditions. Culture medium was then replaced with cRPMI and incubated for 17 h under standard culture conditions. Cell-free supernatants were then harvested, centrifuged for 3 min at 10,000 x g at 4 °C and stored at −80 °C prior to cytokine detection.

### Cytokine quantification

Cytokine quantification was conducted using a ProcartaPlex multiplex immunoassay following the manufacturers recommendations (Thermo Fischer Scientific, Waltham, MA, USA). Briefly, 50 µl of cell-free co-culture supernatants (CFS) were used for cytokine quantification using a MAGPIX® MILLIPLEX® system (Merck, Darmstadt, Germany) with the xPONENT software (Luminex, Austin, TX, USA). Data was analysed using the MILLIPLEX® analyst software (Merck) using a 5-parameter logistic curve and background subtraction to convert mean fluorescence intensity to pg/ml values.

### Transcriptional analysis using microarrays

HT29-MTX cells were cultured in 24-well Transwell® (Corning, Corning, NY, USA), and incubated with treatments of MRx0518_LV_, MRx0518_HK_ and MRx0518_SN_ at a MOI of 100:1 (or equivalent) for 3 h at 37 °C under anaerobic conditions. Cells were washed and lysed, and RNA was isolated from lysate using an RNeasy Mini Kit (Qiagen, Hilden, Germany). RNA was converted to cDNA using a GeneChip™ High Throughput WT PLUS Kit, which was then hybridized to a GeneChip™ Human Transcriptome Array 2.0. Microarray chips were washed and stained using a GeneChip™ Fluidics Station 450 instrument and the GeneChip™ Expression Wash, Stain and Scan kit, and then scanned using a GeneChip™ Scanner 3000 instrument (Thermo Fischer Scientific). Data analysis was carried out using Transcriptome Analysis Console 4.0 software (Thermo Fischer Scientific). Data were normalized using the Robust Multiarray Average algorithm, and fold changes were calculated using the normalized log_2_-transformed values of treated cells relative to respective controls. Data were filtered using cut-offs of *p* < 0.05, fold change of <−1.5 and ≥1.5, and the presence of a gene symbol and coding variants. Pathway analysis was carried out using MetaCore™ (Clarivate Analytics, Philadelphia, PA, USA).

### NF-κB and TLR5 reporter assays

THP1-Blue™ NF-kB and HEK-Blue™-hTLR5 cells (InvivoGen, San Diego, CA, USA), grown to 90% density were washed once with phosphate-buffered saline (PBS) (Sigma-Aldrich, Gillingham, England, UK) and resuspended in growth media without antibiotic at a density of 160,000 and 500,000 cells/ml, respectively. MRx0518_LV_ was added at a MOI of 10:1, MRx0518_HK_ was used at a MOI of 100:1 and a 100:1 MOI equivalent volume was used for the supernatant fractions. Recombinant proteins were added at concentrations of 0.006–500 ng/ml. Positive controls for each reporter assay, *Salmonella* Typhimurium flagellin (FLA-ST) and heat-killed *L. monocytogenes* (HKLM) (InvivoGen), were used at 20 ng/ml concentrations and a MOI of 200:1 respectively. Cells were then incubated at 37 °C in a 5% CO_2_ atmosphere for 22 h. QUANTI-Blue™ (InvivoGen) was added to cells, plates were incubated for a further 2 h and the optical density at 655 nm was recorded. Graphs show results from averaged technical replicates and at least three independent experiments.

### Transcriptional analysis of the flagellar loci of *E. gallinarum* MRx0518

Total RNA was extracted from late-log phase cultures of strain MRx0518, treated with RNAprotect (Qiagen), using the RNeasy Mini kit (Qiagen) according to the manufacturer’s protocol with minor modifications. Briefly, mechanical cell lysis was performed using Lysing Matrix B and a MP Fast-Prep-24 tissue and cell homogenizer (MP Biomedicals, Santa Ana, CA, USA) with oscillations set at 6 m/s. Cells were disrupted for two 20 s cycles with a 1 min rest on ice between cycles. RNA quality was assessed using a Tapestation (Agilent Technologies, Santa Clara, CA, USA) with the Agilent RNA Screentape (Agilent Technologies). The absence of RNA degradation was confirmed, and all samples had a minimum RNA Integrity Numbers ≥ 9. MICROB*Express* kit (Thermo Fischer Scientific) was used to deplete rRNA species and the absence of 16 S and 23 S rRNA species was assessed using an Agilent Tapestation with the Agilent RNA Screentape (Agilent Technologies). RNA samples depleted in rRNA were sent to GATC Biotech for strand-specific library preparation and Illumina sequencing was performed to produce 50 bp single-end reads. An average of 18,705,633 raw reads were generated per RNA-Seq library. Raw reads were trimmed using Trimmomatic^51^ and quality filtered (an average of 18,245,365.6 reads/library passed QC) reads were aligned (99.05% of total clean reads mapped) to the MRx0518 genome using Bowtie^52^. Data generated from three biological replicates were merged using BAMtools^53^ and subsequently used to calculate the expression levels of the motility loci of strain MRx0518 using Geneious R11 (Biomatters, Auckland, New Zealand). The read numbers associated with each gene were expressed in RPKM (reads per kilobases per million reads) scores^54^.

### Motility assays

Motility *in vitro* was assessed using BBL™ Motility Test Medium supplemented with 0.005% (w/v) 2,3,5-triphenyltetrazolium chloride (BD, Sparks, MD, USA). In brief, a fresh colony was stab-inoculated in 20 ml equilibrated media and incubated for 48 h at 37 °C in anaerobic conditions. All assays were performed in triplicate.

### Protein identification by nanoLC-MS/MS

Sample preparation and protein identification by LC-MS/MS were performed by Aberdeen Proteomics (University of Aberdeen, UK). In brief, 40 ml culture supernatants were concentrated down to 0.5 ml and washed with ultrapure water. Proteins were precipitated using a ReadyPrep 2-D Cleanup Kit (Bio-Rad) and resuspended in 100 µl 50 mM ammonium bicarbonate. Proteins were incubated with porcine trypsin (Promega, Madison, WI, USA) for 16 h at 37 °C and the resulting supernatants were dried by vacuum centrifugation and dissolved in 0.1% trifluoroacetic acid. Peptides were further desalted using µ-C18 ZipTips (Merck). Peptides were then eluted into a 96-well microtiter plate, dried by vacuum centrifugation and dissolved in 10 µl LC-MS loading solvent (2% acetonitrile, 0.1% formic acid). Peptides were separated and identified by nanoLC-MS/MS (Q Exactive hybrid quadrupole-Orbitrap MS system) (Thermo Fischer Scientific) using a 15-cm PepMap column, 60-minute LC-MS acquisition method and an injection volume of 5 µl. Data analysis was performed with Proteome Discoverer (Thermo Fischer Scientific) and the workflow included the Mascot Server as the search engine with the following parameters: enzyme = trypsin, maximum mixed cleavage sites = 2, precursor mass tolerance = 10 ppm, dynamic modifications = oxidation (M), static modifications = carbamidomethyl (C). Identified peptides were matched against a strain-specific protein sequence database, which was constructed based on the sequenced genome of *E. gallinarum* MRx0518 (3068 sequences).

### Recombinant flagellin expression and purification

*E. gallinarum* MRx0518 and DSM100110 full-length *fliC* genes were amplified by PCR using primer pairs DC022/DC023 and DC024/DC025, respectively (Supplementary Table S3). Gene products were then cloned into the pQE-30 vector (Supplementary Table S3) (Qiagen) using *Bam*HI and *Sal*I restriction sites. The resulting constructs, which add 12 amino acid residues (MRGSHHHHHHGS) to the N-terminal end of the proteins, were then transformed into *E. coli* M15 pREP4 (Supplementary Table S3) (Qiagen) for over-expression. Expression of recombinant proteins were induced according to the manufacturer’s instructions, by adding 0.1 mM IPTG for 18 h at 20 °C with shaking (200 rpm). *E. coli* cells were lysed by sonication and the recombinant proteins were purified using Ni-NTA columns (Qiagen). An empty vector control was also expressed and purified in parallel, to provide a control for the potential effect of residual contaminants and endotoxins. Endotoxins were removed using Pierce™ High Capacity Endotoxin Removal Spin Column (Thermo Fischer Scientific) according to the manufacturer’s instructions. Residual endotoxin levels were quantified using Pierce™ LAL Chromogenic Endotoxin Quantification Kit (Thermo Fischer Scientific) and shown to be suitable for co-culture assays (Supplementary Fig. S3). Protein concentrations were measured using Pierce™ BCA Protein Assay Kit (Thermo Fischer Scientific) and the purity of each recombinant protein preparations was assessed by SDS-PAGE (Bio-Rad, Hercules, CA, USA) (Supplementary Fig. S3).

### Sequencing and annotation of the flagellar loci and *fliC* genes of *E. gallinarum* and *E. casseliflavus* strains

The flagellar loci of *E. gallinarum* strains MRx0518 and DSM100110 and the *fliC* genes of *E. gallinarum* and *E. casseliflavus* were sequenced as part of ongoing bacterial genome sequencing projects carried out by Diversigen (Houston, TX, USA), GATC Biotech (Konstanz, Germany) and MicrobesNG (Birmigham, England, UK) on behalf of 4D pharma Research Ltd (for additional details see Supplementary Table S3). MicrobesNG (http://www.microbesng.uk) is supported by the BBSRC (grant number BB/L024209/1). The “Rapid Annotation using Subsystem Technology” (RAST) database was used for automated annotation of open reading frames^55–57^ followed by manual curation of the gene annotations in Geneious R11. The flagellar locus and *fliC* of strain MRx0518 was used as a reference sequence for all comparative analyses and homologs (as determined by BLASTp similarity searches) within additional strains were identified and extracted from the draft genomes of available *E. gallinarum* or *E. casseliflavus* genomes downloaded from NCBI (https://www.ncbi.nlm.nih.gov/genome/).

### Comparative analysis of the flagellar loci of *E. gallinarum* strains MRx0518 and DSM100110

Nucleotide alignments were generated using a local BLAST v 2.7.1+ installation which were then visualised and analysed for gene conservation and sequence synteny using EasyFig. 2.2.2^58^.

### Phylogenetic analyses

FliC protein sequences were downloaded from the NCBI protein database or were derived from sequence data available for the strains outlined in Table S5, using BLASTP-based homology searches against the FliC_MRx0518_ sequence. Protein sequences were aligned using MUSCLE^59^ and evolutionary analyses were conducted in MEGA7^60^. Phylogenies were inferred using the Maximum Likelihood method based on the Le_Gascuel_2008 model^61^. A discrete Gamma distribution was used for the multispecies FliC tree (Fig. S5), to model evolutionary rate differences among sites (5 categories (+G)). The rate-variation model allowed for some sites to be evolutionarily invariable ([+I]). The trees with the highest log likelihood are displayed and the reliability of the groups were evaluated by bootstrap testing with 1,000 re-samplings. The FliC of *Lactobacillus mali* DSM20444 (accession number KRN11091.1) was used as an outgroup during *E. gallinarum* and *E. casseliflavus* interspecies phylogenetic analyses.

### Generation of an *E. gallinarum* MRx0518 flagellin gene insertion mutant

The flagellin insertion mutant was created using the non-replicative plasmid pORI19^30^ (Supplementary Table S3). An internal fragment of *E. gallinarum* MRx0518 *fliC* gene was amplified using primers DC020 and DC021 (Supplementary Table S3) and cloned into pORI19. Restriction enzymes and Quick Ligase (New England Biolabs, Ipswich, MA, USA) were used according to the manufacturer’s instructions. This construct was propagated in *E. coli* EC101 by chemical transformation (Supplementary Table S3) and isolated using the Genopure Plasmid Maxi Kit (Roche Diagnostics, Basel, Switzerland) from a 500-ml culture. Isolated plasmid DNA was concentrated using 0.3 M sodium acetate pH 5.2 and ethanol down to 20 μl. A protocol was developed to prepare *E. gallinarum* MRx0518 electrocompetent cells, which was adapted from a previously published method^29^. In brief, *E. gallinarum* MRx0518 was grown for 18 h in GM17 broth, supplemented with 0.5 M sucrose and 3% (w/v) glycine (Sigma-Aldrich). Cells were then washed twice with 0.5 M sucrose and 10% (v/v) glycerol and treated with 10 μg/ml lysozyme and 10 U/ml mutanolysin (Sigma-Aldrich) for 30 min at 37 °C. *E. gallinarum* MRx0518 cells were then transformed by electroporation with 10 μg of plasmid DNA and recovered in BHI broth before plating on selective BHI agar. Plasmid insertion was confirmed for successful transformants by PCR amplification and sequencing (GATC Biotech, Konstanz, Germany) using primers listed in Supplementary Table S3. *In vitro* motility of the flagellin insertion mutant was assessed as described for strain MRx0518.

### Gene expression profiling by qPCR

HT29-MTX cells were cultured in Transwell® and incubated with bacterial culture supernatant (MOI 100:1 equivalent) or recombinant flagellin (1 µg/ml) for 24 h. Mammalian RNA was isolated as described above. cDNA was synthesized using a High-Capacity cDNA Reverse Transcription Kit (Thermo Fischer Scientific). qPCR analysis was carried out using the primers detailed in Supplementary Table S3 and Power SYBR™ Green PCR Master Mix (Thermo Fischer Scientific).

### IL-8 ELISA

IL-8 secretion was quantified from HT29-MTX co-culture supernatants after 24 h of treatment (with bacterial culture supernatant at a MOI 100:1 equivalent or 1 µg/ml recombinant flagellin) using the Human IL-8 (CXCL8) standard ABTS ELISA development kit (Peprotech, Rocky Hill, NJ, USA) according to the manufacturer’s instructions.

## Supplementary information

SREP-18-27269A Supplementary Information

SREP-18-27269A Supplementary Dataset

## Data Availability

The motility loci of *E. gallinarum* MRx0518 and DSM100110 have been deposited under GenBank accession numbers MK210233 and MK176551, respectively. The *fliC* genes of the *E. gallinarum* and *E. casseliflavus* strains outlined in Supplementary Table S3 have been deposited under GenBank accession numbers MK142539-MK142553 and MK174384- MK174386 respectively. Raw RNA-Seq reads are available at the Sequence Read Archive (SRA) under BioProject accession number: PRJNA506224. Microarray data were submitted to the National Center for Biotechnology Information into the Gene Expression Omnibus (GEO) database under accession number GSE122232.
