# Supplementary material for: The flagellin of candidate live biotherapeutic Enterococcus gallinarum MRx0518 is a potent immunostimulant
Source: Sci Rep. 2019 Jan 28;9:801. doi: 10.1038/s41598-018-36926-8 (PMC6349862; doi:10.1038/s41598-018-36926-8)
Supplement: Supplementary file 1 — SREP-18-27269A Supplementary Information [file 41598_2018_36926_MOESM1_ESM.pdf]

The flagellin of candidate live biotherapeutic *Enterococcus gallinarum* MRx0518 is a potent immunostimulant.

Delphine L. Lauté-Caly[a,#], Emma J. Raftis[a,*,#], Philip Cowie[a], Emma Hennessy[a], Amy Holt[a], D. Alessio Panzica[a], Christina Sparre[a], Beverley Minter[a], Eline Stroobach[a] and Imke E. Mulder[a]

Affiliation:

[a] 4 D Pharma Research Ltd, Life Science Innovation Building, Cornhill Road, Aberdeen, AB25 2ZS, United Kingdom.

[#]D.L.C. and E.J.R. contributed equally to this work.

***Corresponding author: emma.raftis@4dpharmaplc.com.**

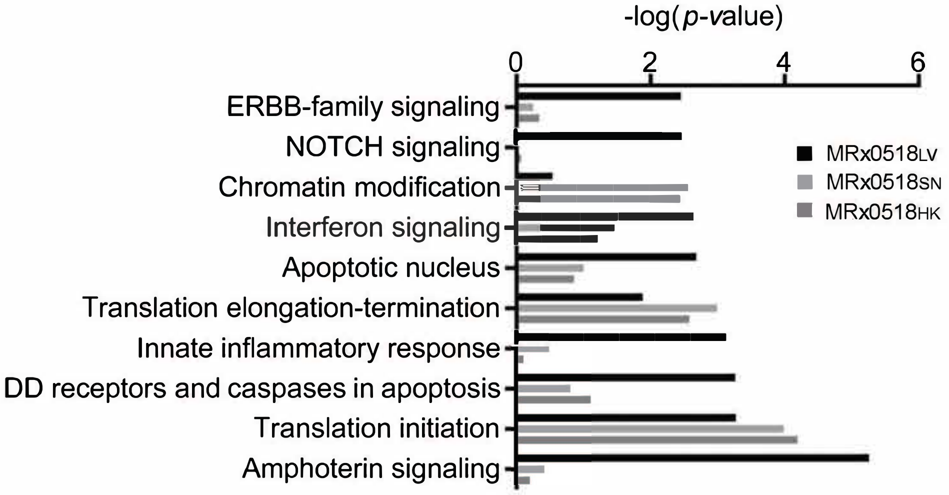

Figure S1. Metacore pathway analysis. Upregulated process networks calculated using Metacore (Clarivate Analytics) from the transcriptomic analysis of HT29-MTX cells response to MRx0518 treatments after 3 h co-culture (MOI 100:1).

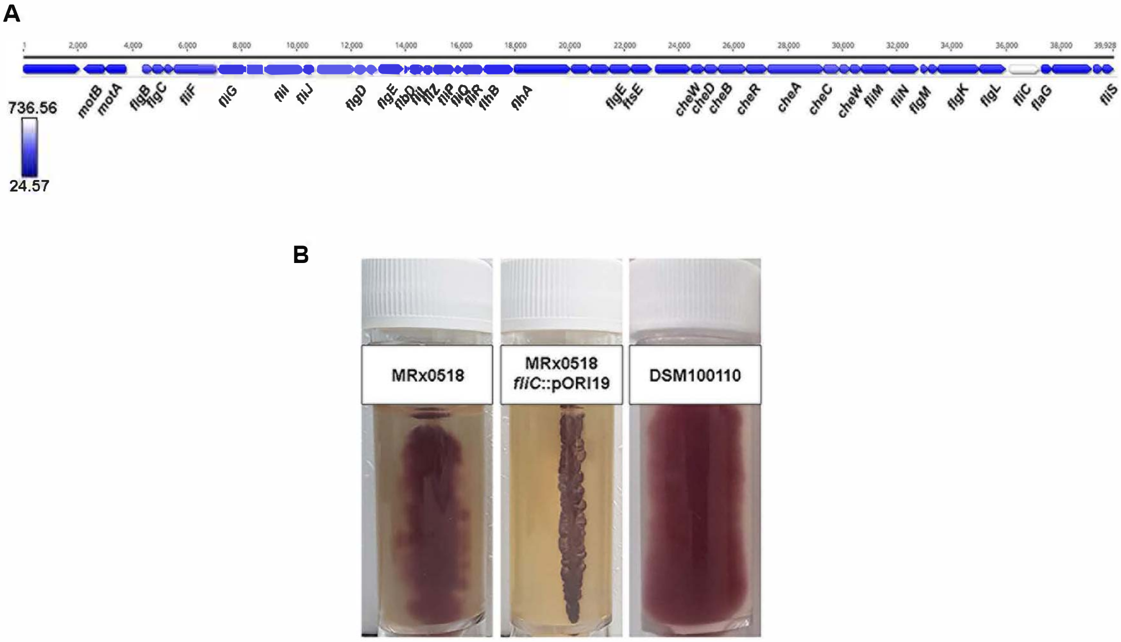

Figure S2. Analysis of *E. gallinarum* MRx0518 motility. A. Transcriptional analysis of the flagellar loci genes of *E. gallinarum* MRx0518 during late log phase growth. The relative Reads Per Kilobase of transcript per Million mapped reads (RPKM) of each gene in the region is indicated according to the colour scale. **B.** Motility of *E. gallinarum* MRx0518, MRx0518 *fliC*::pORI19 and DSM100110 *in vitro*, assessed on soft agar after 48 h incubation at 37°C under anaerobic conditions.

A

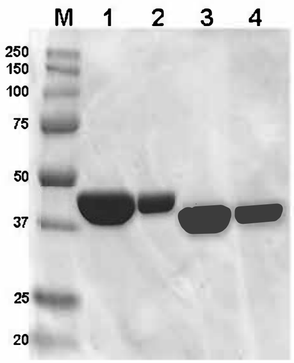

B

	FliC$_{MRx0518}$	FliC$_{DSM100110}$
Protein Concentration (mg/ml)	0.625	0.626
Endotoxin Concentration (EU/ml)	0.880	0.990

Figure S3. Qualitative assessment of purified recombinant proteins FliC$_{MRx0518}$ and FliC$_{DSM100110}$. A. SDS-PAGE analysis (12% acrylamide gel). Lane M: 5 µl Bio-Rad All Blue Prestained Protein Standards, lane 1: 6.25 µg FliC$_{MRx0518}$, lane 2: 6.25 µg FliC$_{DSM100110}$. **B.** Protein and entotoxin concentrations of the purified recombinant protein samples.

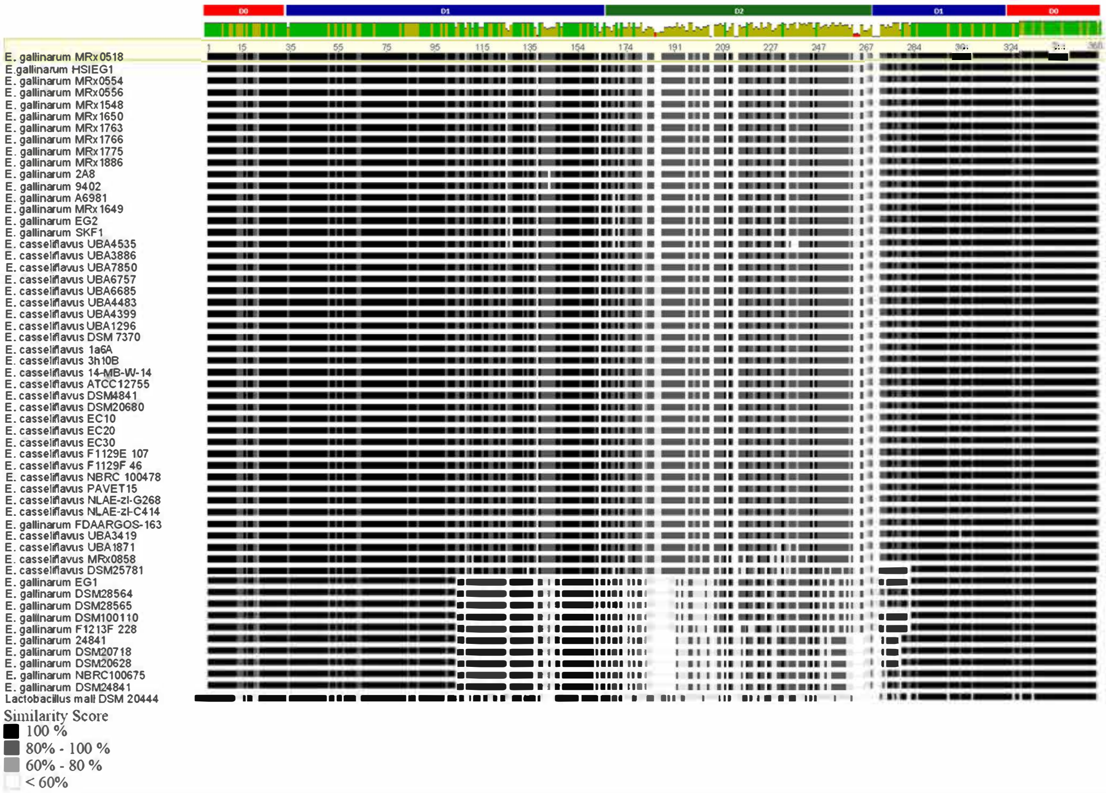

Figure S4. Illustration of the amino acid sequence alignments of the FliC proteins of *E. gallinarum* and *E. casseliflavus* strains. BLOSUM62 was used as the substitution matrix used for the alignments. The mean pairwise identity level across the protein and between strains are indicated above the alignment as follows: green = 100%; brown = between 30% and 100% and red = less than 30%. Predicted domains D0 (D0:2-32 and 324-358,), D1 (33-164 and 273-317) and D2 (165-272) are indicated above the alignment. Amino acid level similarity is indicated by the accompanying legend.

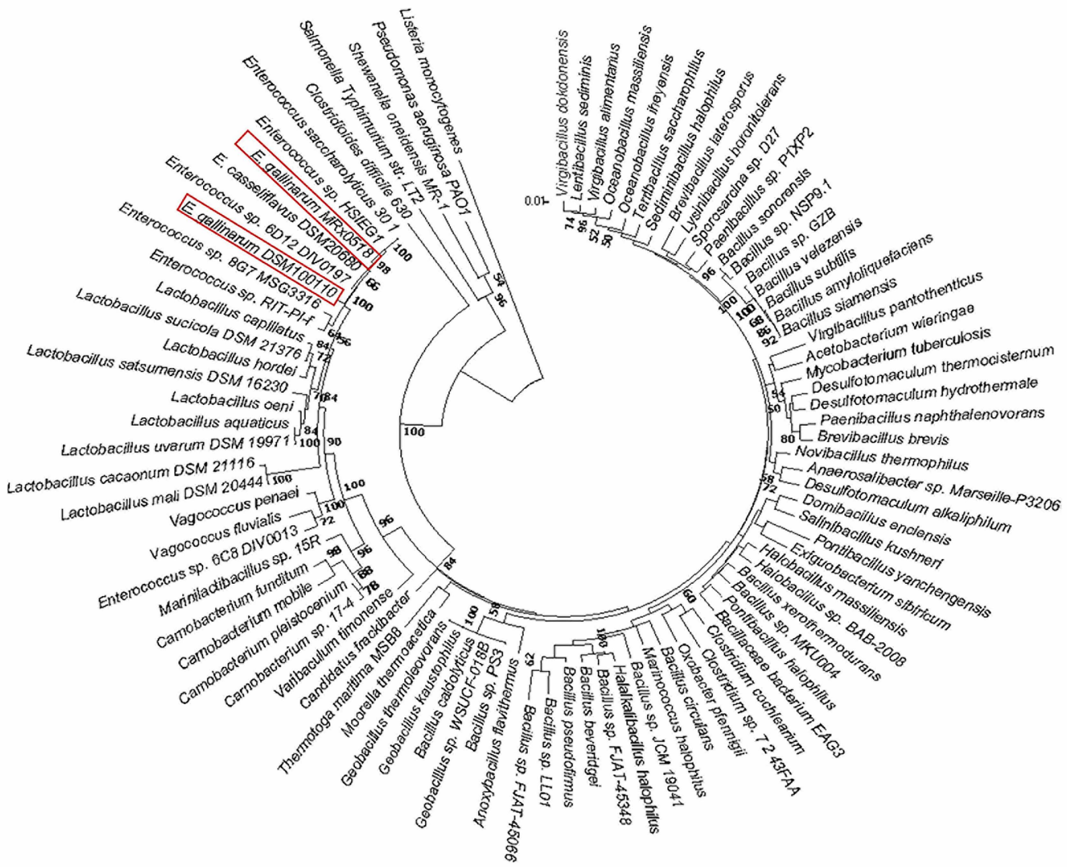

Figure S5. Phylogenetic analysis of multispecies FliC proteins which display homology to FliC$_{MRx0518}$. The sequences were aligned with MUSCLE, and the trees were inferred with MEGA7 software. The evolutionary history was inferred by using the maximum likelihood method based on the LG +G +I model. Statistical support (above 50%) was estimated with bootstraps and is indicated at branch nodes. Homologs were determined by BLASTP searches against the Landmark database. In addition, the FliC proteins of *L. monocytogenes* strain, *P. aeruginosa* PAO1 and *C. difficile* strain were included as reference sequences, as were the FliC$_{DSM100110}$ and FliC$_{DSM20680}$.

Table S1. List of up- and down-regulated genes in HT29-MTX cells after 3 h contact with *E. gallinarum* MRx0518 live cells (MRx0518$_{LV}$), heat-killed (MRx0518$_{HK}$) or culture supernatants (MRx0518$_{SN}$) (MOI 100:1) (n=3). Genes were filtered based on a fold change of ≥ 1.5 or ≤ -1.5 and a *p*-value < 0.05, on coding transcripts only and on the presence of a gene symbol.

		MRx0518$_{LV}$		MRx0518$_{HK}$		MRx0518$_{SN}$	
**Gene symbol**	**Description**	**Fold change[a]**	***p*-value**	**Fold change[a]**	***p*-value**	**Fold change[a]**	***p*-value**
ACBD3	Acyl-CoA binding domain containing 3	n.s.	n.s.	n.s.	n.s.	1.54	0.0003
ACTR3	ARP3 actin-related protein 3 homolog (yeast)	n.s.	n.s.	n.s.	n.s.	1.59	0.0043
ADGRF1	Adhesion G protein-coupled receptor F1	n.s.	n.s.	n.s.	n.s.	1.72	0.034
ADGRG6	Adhesion G protein-coupled receptor G6	n.s.	n.s.	n.s.	n.s.	1.59	0.0086
ADM	Adrenomedullin	1.54	8.14E-06	n.s.	n.s.	n.s.	n.s.
AGA	Aspartylglucosaminidase	n.s.	n.s.	1.54	0.0026	n.s.	n.s.
AHCTF1P1	AT hook containing transcription factor 1 pseudogene 1	2.17	0.0414	n.s.	n.s.	n.s.	n.s.
AKAP9	A kinase (PRKA) anchor protein 9	1.57	0.0203	n.s.	n.s.	n.s.	n.s.
ANKRD18CP	Ankyrin repeat domain 18C, pseudogene	n.s.	n.s.	n.s.	n.s.	1.54	0.0063
ANTXR2	Anthrax toxin receptor 2	n.s.	n.s.	n.s.	n.s.	1.51	0.0238
APOBEC1	Apolipoprotein B mRNA editing enzyme, catalytic polypeptide 1	2.05	7.56E-07	1.76	3.28E-06	2.27	1.16E-07
APOH	Apolipoprotein H (beta-2-glycoprotein I)	n.s.	n.s.	1.58	0.0199	1.69	0.0007
APPBP2	Amyloid beta precursor protein (cytoplasmic tail) binding protein 2	n.s.	n.s.	n.s.	n.s.	1.63	0.046
APPL2	Adaptor protein, phosphotyrosine interaction, PH domain and leucine zipper containing 2	n.s.	n.s.	n.s.	n.s.	1.58	0.0063
APRT	Adenine phosphoribosyltransferase	n.s.	n.s.	1.55	0.0185	n.s.	n.s.
APTR	Alu-mediated CDKN1A/p21 transcriptional regulator (non-protein coding)	1.84	1.05E-07	n.s.	n.s.	n.s.	n.s.
AREL1	Apoptosis resistant E3 ubiquitin protein ligase 1	n.s.	n.s.	n.s.	n.s.	1.66	0.0268
ARFGAP3; PACSIN2	ADP-ribosylation factor GTPase activating protein 3; protein kinase C and casein kinase substrate in neurons 2	n.s.	n.s.	n.s.	n.s.	1.68	0.0193
ARHGEF38	Rho guanine nucleotide exchange factor 38	1.53	0.0017	n.s.	n.s.	n.s.	n.s.
ARL14	ADP-ribosylation factor like GTPase 14	n.s.	n.s.	n.s.	n.s.	1.73	0.0166
ARL5B	ADP-ribosylation factor like GTPase 5B	1.62	0.0448	n.s.	n.s.	1.54	0.0145
ARL6IP5	ADP-ribosylation factor like GTPase 6 interacting protein 5	n.s.	n.s.	n.s.	n.s.	1.94	0.0094
ARMC1	Armadillo repeat containing 1	1.53	0.0281	n.s.	n.s.	1.59	0.0377
ARNT	Aryl hydrocarbon receptor nuclear translocator	n.s.	n.s.	n.s.	n.s.	1.53	0.049
ARRDC3	Arrestin domain containing 3	3.65	0.0002	n.s.	n.s.	n.s.	n.s.
ARRDC4	Arrestin domain containing 4	1.7	0.0244	n.s.	n.s.	n.s.	n.s.
ASB8	Ankyrin repeat and SOCS box containing 8	n.s.	n.s.	n.s.	n.s.	1.62	0.0304
ASF1A	Anti-silencing function 1A histone chaperone	1.57	0.0092	n.s.	n.s.	n.s.	n.s.
ASNS	Asparagine synthetase (glutamine-hydrolyzing)	n.s.	n.s.	n.s.	n.s.	1.51	0.0028
ATF3	Activating transcription factor 3	2.09	7.27E-05	n.s.	n.s.	n.s.	n.s.
ATP6V1C1	ATPase, H+ transporting, lysosomal 42kDa, V1 subunit C1	n.s.	n.s.	n.s.	n.s.	1.75	0.0318
B4GALT5	UDP-Gal:betaGlcNAc beta 1,4- galactosyltransferase, polypeptide 5	n.s.	n.s.	n.s.	n.s.	1.77	0.0091
BCAS1	Breast carcinoma amplified sequence 1	n.s.	n.s.	n.s.	n.s.	1.73	0.0248
BCAS2	Breast carcinoma amplified sequence 2	n.s.	n.s.	1.54	0.0127	n.s.	n.s.
BIRC3	Baculoviral IAP repeat containing 3	2.3	3.56E-07	n.s.	n.s.	1.64	6.45E-05
BOLA1	BolA family member 1	n.s.	n.s.	1.56	0.0002	n.s.	n.s.
BRAF	B-Raf proto-oncogene, serine/threonine kinase	n.s.	n.s.	n.s.	n.s.	1.54	0.0025
BRI3	Brain protein I3	-1.64	0.003	n.s.	n.s.	n.s.	n.s.
BRK1	BRICK1, SCAR/WAVE actin-nucleating complex subunit	1.57	0.0346	1.65	0.0085	n.s.	n.s.
BROX	BRO1 domain and CAAX motif containing	1.84	0.0365	n.s.	n.s.	n.s.	n.s.
BRWD3	Bromodomain and WD repeat domain containing 3	n.s.	n.s.	n.s.	n.s.	1.65	0.041
BTBD3	BTB (POZ) domain containing 3	n.s.	n.s.	n.s.	n.s.	1.56	0.0429

BX571672.3	MicroRNA 3118-2	-1.55	0.0243	n.s.	n.s.	n.s.	n.s.
C11ORF49	Chromosome 11 open reading frame 49	n.s.	n.s.	n.s.	n.s.	1.71	0.0448
C11ORF71	Chromosome 11 open reading frame 71	n.s.	n.s.	1.8	0.0343	n.s.	n.s.
C14ORF1	Chromosome 14 open reading frame 1	1.52	0.0013	1.57	0.0017	n.s.	n.s.
C15ORF65	Chromosome 15 open reading frame 65	n.s.	n.s.	1.7	0.0162	n.s.	n.s.
C2ORF15	Chromosome 2 open reading frame 15	n.s.	n.s.	-1.77	0.0373	n.s.	n.s.
C5ORF51	Chromosome 5 open reading frame 51	n.s.	n.s.	n.s.	n.s.	1.53	0.0171
C8ORF76	Chromosome 8 open reading frame 76	n.s.	n.s.	1.79	0.0431	n.s.	n.s.
CAP1	CAP, adenylate cyclase-associated protein 1 (yeast)	n.s.	n.s.	n.s.	n.s.	1.7	0.012
CASP4	Caspase 4	1.55	0.0157	1.55	0.0073	n.s.	n.s.
CCDC28A	Coiled-coil domain containing 28A	n.s.	n.s.	n.s.	n.s.	1.5	7.84E-06
CCL20	Chemokine (C-C motif) ligand 20	20.59	1.10E-13	n.s.	n.s.	8.22	2.06E-11
CDC5L	Cell division cycle 5-like	1.61	0.0045	n.s.	n.s.	1.66	0.0002
CDH17	Cadherin 17, LI cadherin (liver-intestine)	n.s.	n.s.	n.s.	n.s.	2.26	0.0358
CDKN1B	Cyclin-dependent kinase inhibitor 1B (p27, Kip1)	1.59	0.002	n.s.	n.s.	n.s.	n.s.
CDR2	Cerebellar degeneration related protein 2	1.75	0.0067	n.s.	n.s.	n.s.	n.s.
CEACAM1	Carcinoembryonic antigen-related cell adhesion molecule 1 (biliary glycoprotein)	n.s.	n.s.	n.s.	n.s.	1.58	0.0351
CEACAM5	Carcinoembryonic antigen-related cell adhesion molecule 5	n.s.	n.s.	n.s.	n.s.	1.95	0.0372
CEACAM7	Carcinoembryonic antigen-related cell adhesion molecule 7	n.s.	n.s.	n.s.	n.s.	2.35	0.0064
CEP57	Centrosomal protein 57kDa	1.51	0.0098	n.s.	n.s.	n.s.	n.s.
CEP97	Centrosomal protein 97kDa	1.51	0.0148	n.s.	n.s.	n.s.	n.s.
CIB1	Calcium and integrin binding 1 (calmyrin)	n.s.	n.s.	1.53	0.0007	n.s.	n.s.
CLINT1	Clathrin interactor 1	n.s.	n.s.	n.s.	n.s.	1.71	0.0029
CLOCK	Clock circadian regulator	n.s.	n.s.	n.s.	n.s.	1.54	0.0376
CLPP	Caseinolytic mitochondrial matrix peptidase proteolytic subunit	1.63	0.0037	n.s.	n.s.	n.s.	n.s.
COG5	Component of oligomeric golgi complex 5	n.s.	n.s.	n.s.	n.s.	1.6	0.0344
CREB3	CAMP responsive element binding protein 3	1.51	0.0249	n.s.	n.s.	n.s.	n.s.
CRKL	V-crk avian sarcoma virus CT10 oncogene homolog-like	1.81	0.0018	1.56	0.003	n.s.	n.s.
CRNKL1	Crooked neck pre-mRNA splicing factor 1	n.s.	n.s.	n.s.	n.s.	1.52	0.0011
CRYZ	Crystallin zeta	n.s.	n.s.	n.s.	n.s.	1.5	0.0025
CST9L	Cystatin 9-like	-1.55	0.0245	n.s.	n.s.	n.s.	n.s.
CTAGE4; CTAGE8	CTAGE family, member 4; CTAGE family, member 8	n.s.	n.s.	n.s.	n.s.	1.66	0.0118
CTBS	Chitobiase, di-N-acetyl-	n.s.	n.s.	n.s.	n.s.	1.91	0.0256
CTCF	CCCTC-binding factor (zinc finger protein)	n.s.	n.s.	n.s.	n.s.	1.52	0.0287
CTH	Cystathionine gamma-lyase	n.s.	n.s.	n.s.	n.s.	1.73	0.0127
CTTNBP2NL	CTTNBP2 N-terminal like	n.s.	n.s.	n.s.	n.s.	1.58	0.0477
CUL1	Cullin 1	n.s.	n.s.	n.s.	n.s.	1.57	0.0001
CWC15	CWC15 spliceosome-associated protein	n.s.	n.s.	1.52	0.0282	n.s.	n.s.
CXCL1	Chemokine (C-X-C motif) ligand 1 (melanoma growth stimulating activity, alpha)	2.41	1.13E-08	n.s.	n.s.	n.s.	n.s.
CXCL2	Chemokine (C-X-C motif) ligand 2	1.64	2.27E-05	n.s.	n.s.	n.s.	n.s.
CXCL8	Chemokine (C-X-C motif) ligand 8	4.87	5.24E-13	n.s.	n.s.	1.56	3.03E-05
CYP1B1	Cytochrome P450, family 1, subfamily B, polypeptide 1	1.56	0.0134	1.57	0.0115	n.s.	n.s.
CYP3A5	Cytochrome P450, family 3, subfamily A, polypeptide 5	1.68	0.019	n.s.	n.s.	n.s.	n.s.
DAAM1	Dishevelled associated activator of morphogenesis 1	n.s.	n.s.	n.s.	n.s.	1.56	0.0212
DCXR	Dicarbonyl/L-xylulose reductase	n.s.	n.s.	n.s.	n.s.	1.79	0.03
DDIT3	DNA-damage-inducible transcript 3	2.22	0.0028	1.76	0.0146	n.s.	n.s.
DDIT4	DNA damage inducible transcript 4	2.51	0.0007	n.s.	n.s.	n.s.	n.s.
DDX24	DEAD (Asp-Glu-Ala-Asp) box helicase 24	n.s.	n.s.	n.s.	n.s.	1.92	0.0373
DHRS4-AS1	DHRS4 antisense RNA 1	n.s.	n.s.	n.s.	n.s.	1.56	0.0025

DHRS9	Dehydrogenase/reductase (SDR family) member 9	n.s.	n.s.	n.s.	n.s.	2.26	0.0119
DIEXF	Digestive organ expansion factor homolog (zebrafish)	n.s.	n.s.	n.s.	n.s.	1.63	0.0226
DNAJC3-AS1	DNAJC3 antisense RNA 1 (head to head)	1.58	0.0025	n.s.	n.s.	n.s.	n.s.
DPF2	D4, zinc and double PHD fingers family 2	1.58	0.0262	n.s.	n.s.	n.s.	n.s.
DPY30	Dpy-30 histone methyltransferase complex regulatory subunit	n.s.	n.s.	1.62	0.0028	1.65	0.0005
DRAM2	DNA-damage regulated autophagy modulator 2	1.55	6.40E-05	1.56	8.39E-05	1.52	1.44E-05
DUOXA2	Dual oxidase maturation factor 2	1.75	4.65E-05	n.s.	n.s.	n.s.	n.s.
DUSP6	Dual specificity phosphatase 6	1.6	5.44E-05	n.s.	n.s.	n.s.	n.s.
DYNC1LI2	Dynein, cytoplasmic 1, light intermediate chain 2	n.s.	n.s.	n.s.	n.s.	1.76	0.0496
EBLN3	Endogenous Bornavirus-like nucleoprotein 3	n.s.	n.s.	n.s.	n.s.	1.95	0.0286
EBPL	Emopamil binding protein-like	n.s.	n.s.	1.53	0.032	n.s.	n.s.
EDF1	Endothelial differentiation-related factor 1	1.6	0.0005	1.7	3.60E-05	1.67	0.0001
EFTUD1	Elongation factor Tu GTP binding domain containing 1	n.s.	n.s.	n.s.	n.s.	1.57	0.0234
EIF2S2	Eukaryotic translation initiation factor 2, subunit 2 beta, 38kDa	n.s.	n.s.	n.s.	n.s.	1.55	0.0019
EIF4EBP2	Eukaryotic translation initiation factor 4E binding protein 2	n.s.	n.s.	n.s.	n.s.	1.6	0.0194
ELF3	E74-like factor 3 (ets domain transcription factor, epithelial-specific)	1.53	1.35E-05	n.s.	n.s.	n.s.	n.s.
ELOVL6	ELOVL fatty acid elongase 6	n.s.	n.s.	n.s.	n.s.	1.57	0.0103
EMC4	ER membrane protein complex subunit 4	1.57	0.0014	1.52	0.0015	1.58	0.0039
ENPP1	Ectonucleotide pyrophosphatase/phosphodiesterase 1	n.s.	n.s.	n.s.	n.s.	1.68	0.0199
EPRS	Glutamyl-prolyl-tRNA synthetase	n.s.	n.s.	n.s.	n.s.	1.66	0.0168
EPS15	Epidermal growth factor receptor pathway substrate 15	n.s.	n.s.	n.s.	n.s.	1.62	0.0284
EREG	Epiregulin	2.16	0.0077	n.s.	n.s.	n.s.	n.s.
ERGIC2	ERGIC and golgi 2	n.s.	n.s.	n.s.	n.s.	1.71	0.0002
ESRP1	Epithelial splicing regulatory protein 1	n.s.	n.s.	n.s.	n.s.	1.55	0.0385
EXPH5	Exophilin 5	1.57	0.0412	n.s.	n.s.	n.s.	n.s.
F8A2; F8A3; F8A1	Coagulation factor VIII-associated 2; coagulation factor VIII-associated 3; coagulation factor VIII-associated 1	n.s.	n.s.	n.s.	n.s.	1.64	0.0011
FADD	Fas (TNFRSF6)-associated via death domain	1.51	0.0128	1.57	0.0227	1.8	0.0092
FAM114A1	Family with sequence similarity 114, member A1	n.s.	n.s.	n.s.	n.s.	1.51	0.0115
FAM162A	Family with sequence similarity 162, member A	n.s.	n.s.	1.58	0.0147	1.58	0.0038
FAM175B	Family with sequence similarity 175, member B	1.6	0.01	n.s.	n.s.	1.6	0.0234
FAM177B	Family with sequence similarity 177, member B	n.s.	n.s.	n.s.	n.s.	2.01	0.0005
FAM218A	Family with sequence similarity 218, member A	-1.52	0.0465	n.s.	n.s.	n.s.	n.s.
FAM21A	Family with sequence similarity 21, member A	n.s.	n.s.	n.s.	n.s.	1.52	0.0151
FAM83B	Family with sequence similarity 83, member B	n.s.	n.s.	n.s.	n.s.	1.89	0.0265
FAM96B	Family with sequence similarity 96, member B	1.63	0.0099	1.59	0.0014	n.s.	n.s.
FBXO32	F-box protein 32	n.s.	n.s.	n.s.	n.s.	1.64	0.0068
FBXO8	F-box protein 8	n.s.	n.s.	n.s.	n.s.	1.56	0.0001
FCHO2	FCH domain only 2	n.s.	n.s.	n.s.	n.s.	1.53	0.0107
FEM1C	Fem-1 homolog c (C. elegans)	1.74	0.0056	n.s.	n.s.	n.s.	n.s.
FKBP1AP1	FK506 binding protein 1A pseudogene 1	-1.7	0.0158	n.s.	n.s.	n.s.	n.s.
FKBP2	FK506 binding protein 2	1.7	0.0008	1.75	0.0005	1.77	8.32E-05
FLJ32255	Uncharacterized LOC643977	n.s.	n.s.	n.s.	n.s.	1.86	0.0016
FMO5	Flavin containing monooxygenase 5	n.s.	n.s.	n.s.	n.s.	2.2	0.0293
FNBP1L	Formin binding protein 1-like	1.59	0.01	n.s.	n.s.	n.s.	n.s.
FNTA	Farnesyltransferase, CAAX box, alpha	1.54	0.0002	1.53	0.0008	n.s.	n.s.
FOPNL	FGFR1OP N-terminal like	n.s.	n.s.	n.s.	n.s.	1.63	0.0009
FOXN2	Forkhead box N2	1.72	0.0034	n.s.	n.s.	n.s.	n.s.
G0S2	G0/G1 switch 2	1.55	0.0117	n.s.	n.s.	n.s.	n.s.
GABARAPL2	GABA(A) receptor-associated protein like 2	n.s.	n.s.	n.s.	n.s.	1.53	0.0031
GADD45A	Growth arrest and DNA-damage-inducible, alpha	1.93	0.0001	n.s.	n.s.	n.s.	n.s.

GADD45B	Growth arrest and DNA-damage-inducible, beta	1.73	0.0017	n.s.	n.s.	n.s.	n.s.
GALNT12	Polypeptide N-acetylgalactosaminyltransferase 12	n.s.	n.s.	n.s.	n.s.	1.61	0.0211
GBP3	Guanylate binding protein 3	n.s.	n.s.	n.s.	n.s.	1.66	0.0489
GDF15	Growth differentiation factor 15	1.97	0.013	n.s.	n.s.	1.72	0.0295
GOLM1	Golgi membrane protein 1	n.s.	n.s.	n.s.	n.s.	1.69	0.0432
GOLPH3L	Golgi phosphoprotein 3-like	n.s.	n.s.	n.s.	n.s.	1.62	0.008
GP1BA	Glycoprotein Ib (platelet), alpha polypeptide	-1.57	0.0459	n.s.	n.s.	n.s.	n.s.
GPR89B; GPR89A	G protein-coupled receptor 89B; G protein-coupled receptor 89A	n.s.	n.s.	n.s.	n.s.	-1.51	0.0314
GPX8	Glutathione peroxidase 8 (putative)	n.s.	n.s.	n.s.	n.s.	1.7	0.0173
GRM7-AS2	GRM7 antisense RNA 2	n.s.	n.s.	1.56	0.0173	n.s.	n.s.
GTF2IP1; GTF2IP4	General transcription factor IIi pseudogene 1; general transcription factor IIi, pseudogene 4	n.s.	n.s.	n.s.	n.s.	1.54	0.0282
GTF2IP4; GTF2IP1	General transcription factor IIi, pseudogene 4; general transcription factor IIi pseudogene 1	n.s.	n.s.	n.s.	n.s.	1.53	0.0321
GTF3C2-AS1	GTF3C2 antisense RNA 1	-1.52	0.0006	n.s.	n.s.	n.s.	n.s.
GUCA2B	Guanylate cyclase activator 2B (uroguanylin)	1.83	0.0118	2.18	0.0022	2.35	0.0295
H1F0	H1 histone family, member 0	1.8	2.17E-05	n.s.	n.s.	n.s.	n.s.
H1FX	H1 histone family, member X	n.s.	n.s.	1.53	0.0149	n.s.	n.s.
H3F3C	H3 histone, family 3C	n.s.	n.s.	n.s.	n.s.	1.57	0.0411
HAVCR1	Hepatitis A virus cellular receptor 1	n.s.	n.s.	n.s.	n.s.	2.19	0.0006
HCG27	HLA complex group 27 (non-protein coding)	1.61	0.0075	n.s.	n.s.	n.s.	n.s.
HDAC8	Histone deacetylase 8	n.s.	n.s.	n.s.	n.s.	1.59	0.0348
HIF1A	Hypoxia inducible factor 1, alpha subunit (basic helix-loop-helix transcription factor)	n.s.	n.s.	n.s.	n.s.	1.57	0.0168
HIST1H1E	Histone cluster 1, H1e	2.34	0.0339	n.s.	n.s.	n.s.	n.s.
HIST1H2AB	Histone cluster 1, H2ab	n.s.	n.s.	1.56	0.0088	n.s.	n.s.
HIST1H2AC	Histone cluster 1, H2ac	n.s.	n.s.	1.58	0.0015	n.s.	n.s.
HIST1H2AE	Histone cluster 1, H2ae	n.s.	n.s.	1.74	0.0143	n.s.	n.s.
HIST1H2BD	Histone cluster 1, H2bd	n.s.	n.s.	n.s.	n.s.	1.5	0.0014
HIST1H2BI	Histone cluster 1, H2bi	n.s.	n.s.	1.52	0.0454	n.s.	n.s.
HIST1H2BJ	Histone cluster 1, H2bj	n.s.	n.s.	1.76	0.0071	n.s.	n.s.
HIST1H2BK	Histone cluster 1, H2bk	n.s.	n.s.	n.s.	n.s.	2.42	0.0181
HIST1H3B	Histone cluster 1, H3b	n.s.	n.s.	1.52	0.015	n.s.	n.s.
HIST1H3F	Histone cluster 1, H3f	n.s.	n.s.	1.62	0.0045	n.s.	n.s.
HIST1H3H	Histone cluster 1, H3h	1.68	0.0288	1.78	0.0105	n.s.	n.s.
HIST1H3I	Histone cluster 1, H3i	n.s.	n.s.	2.4	0.0027	n.s.	n.s.
HIST1H3J	Histone cluster 1, H3j	n.s.	n.s.	1.59	0.0051	n.s.	n.s.
HIST1H4C	Histone cluster 1, H4c	n.s.	n.s.	1.78	0.0093	n.s.	n.s.
HIST1H4D	Histone cluster 1, H4d	n.s.	n.s.	1.83	0.0211	n.s.	n.s.
HIST1H4H	Histone cluster 1, H4h	n.s.	n.s.	1.62	0.0029	1.61	0.0023
HIST2H2AB	Histone cluster 2, H2ab	1.59	0.0153	1.75	0.0035	n.s.	n.s.
HIST2H2AC	Histone cluster 2, H2ac	n.s.	n.s.	1.71	0.017	n.s.	n.s.
HIST2H2BE	Histone cluster 2, H2be	n.s.	n.s.	n.s.	n.s.	1.51	0.0096
HIST2H4B; HIST2H4A	Histone cluster 2, H4b; histone cluster 2, H4a	2.59	0.0007	1.67	0.005	n.s.	n.s.
HLA-DMA	Major histocompatibility complex, class II, DM alpha	n.s.	n.s.	n.s.	n.s.	1.64	0.0008
HMGN2P18	High mobility group nucleosomal binding domain 2 pseudogene 18	-1.7	0.0142	n.s.	n.s.	n.s.	n.s.
HNRNPA1P33	Heterogeneous nuclear ribonucleoprotein A1 pseudogene 33	n.s.	n.s.	1.54	0.001	n.s.	n.s.
HOXA6	Homeobox A6	n.s.	n.s.	n.s.	n.s.	1.69	0.012
HSD17B10	Hydroxysteroid (17-beta) dehydrogenase 10	n.s.	n.s.	1.63	0.0172	n.s.	n.s.
HSD17B7P2	Hydroxysteroid (17-beta) dehydrogenase 7 pseudogene 2	1.6	0.0053	n.s.	n.s.	n.s.	n.s.
HSPA1A	Heat shock 70kDa protein 1A	n.s.	n.s.	n.s.	n.s.	1.69	0.0432
HSPB1	Heat shock 27kDa protein 1	1.85	0.0051	n.s.	n.s.	1.52	0.0031

ICAM1	Intercellular adhesion molecule 1	4.03	2.03E-09	n.s.	n.s.	n.s.	n.s.
ICT1	Immature colon carcinoma transcript 1	1.63	0.0377	1.83	0.032	n.s.	n.s.
IDS	Iduronate 2-sulfatase	n.s.	n.s.	n.s.	n.s.	1.57	0.0456
IER3	Immediate early response 3	1.76	0.0082	n.s.	n.s.	n.s.	n.s.
IFIH1	Interferon induced, with helicase C domain 1	1.73	0.0003	n.s.	n.s.	n.s.	n.s.
IFITM2	Interferon induced transmembrane protein 2	n.s.	n.s.	n.s.	n.s.	1.5	0.0033
IFNAR1	Interferon (alpha, beta and omega) receptor 1	n.s.	n.s.	n.s.	n.s.	1.56	1.00E-05
IFRD1	Interferon-related developmental regulator 1	1.52	0.0013	n.s.	n.s.	n.s.	n.s.
IGF2BP2	Insulin-like growth factor 2 mRNA binding protein 2	n.s.	n.s.	n.s.	n.s.	1.71	0.0239
IGLJ5	Immunoglobulin lambda joining 5 (non-functional)	-1.69	0.0134	n.s.	n.s.	n.s.	n.s.
INE1	Inactivation escape 1 (non-protein coding)	-1.52	0.0076	n.s.	n.s.	n.s.	n.s.
IRAK2	Interleukin 1 receptor associated kinase 2	1.51	0.0013	n.s.	n.s.	n.s.	n.s.
JMJD1C	Jumonji domain containing 1C	1.52	0.012	n.s.	n.s.	n.s.	n.s.
JUN	Jun proto-oncogene	1.96	1.48E-07	n.s.	n.s.	n.s.	n.s.
KIAA1033	KIAA1033	n.s.	n.s.	n.s.	n.s.	1.67	0.0199
KIAA1147	KIAA1147	n.s.	n.s.	n.s.	n.s.	1.59	0.0306
KIF3B	Kinesin family member 3B	n.s.	n.s.	n.s.	n.s.	1.52	0.0004
KLF6	Kruppel-like factor 6	1.91	0.0044	n.s.	n.s.	2.1	0.0231
KLHL28	Kelch-like family member 28	1.65	0.0394	n.s.	n.s.	n.s.	n.s.
KMT2E	Lysine (K)-specific methyltransferase 2E	n.s.	n.s.	n.s.	n.s.	1.73	0.0311
KRTCAP3	Keratinocyte associated protein 3	1.52	0.0002	1.57	3.75E-05	n.s.	n.s.
LIMA1	LIM domain and actin binding 1	n.s.	n.s.	n.s.	n.s.	1.85	0.0138
LINC00294	Long intergenic non-protein coding RNA 294	n.s.	n.s.	n.s.	n.s.	1.62	0.0304
LINC00493	Long intergenic non-protein coding RNA 493	n.s.	n.s.	2.22	0.008	n.s.	n.s.
LINC00566	Long intergenic non-protein coding RNA 566	-1.51	0.0192	n.s.	n.s.	n.s.	n.s.
LINC00662	Long intergenic non-protein coding RNA 662	2.09	0.0144	1.59	0.0331	n.s.	n.s.
LINC00667	Long intergenic non-protein coding RNA 667	-1.62	0.0148	n.s.	n.s.	n.s.	n.s.
LINC00673; LINC00511	Long intergenic non-protein coding RNA 673; long intergenic non-protein coding RNA 511	1.92	2.83E-05	1.88	0.0006	n.s.	n.s.
LINC00675	Long intergenic non-protein coding RNA 675	1.57	0.002	n.s.	n.s.	n.s.	n.s.
LINC00696	Long intergenic non-protein coding RNA 696	-1.68	0.0036	n.s.	n.s.	n.s.	n.s.
LINC00837	Long intergenic non-protein coding RNA 837	1.7	0.0446	n.s.	n.s.	n.s.	n.s.
LINC01133	Long intergenic non-protein coding RNA 1133	n.s.	n.s.	n.s.	n.s.	1.59	0.0013
LINC01207	Long intergenic non-protein coding RNA 1207	n.s.	n.s.	n.s.	n.s.	1.67	0.0027
LMLN-AS1	LMLN antisense RNA 1	n.s.	n.s.	n.s.	n.s.	1.52	0.0131
LOC100129034	Uncharacterized LOC100129034	n.s.	n.s.	n.s.	n.s.	1.87	0.0306
LOC100287175	Uncharacterized LOC100287175	n.s.	n.s.	n.s.	n.s.	-1.52	0.0329
LOC100506100	Uncharacterized LOC100506100	n.s.	n.s.	n.s.	n.s.	1.51	0.0112
LOC101927630	Uncharacterized LOC101927630	n.s.	n.s.	n.s.	n.s.	2.47	0.0476
LOC101928694	Uncharacterized LOC101928694	n.s.	n.s.	n.s.	n.s.	-1.5	0.0297
LOC105375166	Uncharacterized LOC105375166	1.68	0.0313	n.s.	n.s.	2.53	0.0063
LOC105377682	Uncharacterized LOC105377682	n.s.	n.s.	-1.59	0.0063	n.s.	n.s.
LOC389765	Kinesin family member 27 pseudogene	1.99	0.0166	n.s.	n.s.	n.s.	n.s.
LOC401589; LOC100129860	Uncharacterized LOC401589; uncharacterized LOC100129860	n.s.	n.s.	n.s.	n.s.	-1.51	0.0393
LOC644919	Uncharacterized LOC644919	n.s.	n.s.	n.s.	n.s.	1.51	0.0171
LRIF1	Ligand dependent nuclear receptor interacting factor 1	n.s.	n.s.	n.s.	n.s.	1.78	0.0099
LRRC31	Leucine rich repeat containing 31	n.s.	n.s.	n.s.	n.s.	1.92	0.0276
LSAMP-AS1	LSAMP antisense RNA 1	n.s.	n.s.	n.s.	n.s.	1.52	0.0288
LSM4	LSM4 homolog, U6 small nuclear RNA and mRNA degradation associated	1.53	0.0214	1.72	0.0413	1.61	0.0491
LXN	Latexin	n.s.	n.s.	1.55	0.0079	n.s.	n.s.
MARCH3	Membrane associated ring finger 3	n.s.	n.s.	n.s.	n.s.	1.51	0.012

MARCKS	Myristoylated alanine-rich protein kinase C substrate	n.s.	n.s.	n.s.	n.s.	1.75	0.0002
MBNL2	Muscleblind-like splicing regulator 2	n.s.	n.s.	n.s.	n.s.	1.76	0.0223
MED21	Mediator complex subunit 21	1.52	0.0006	n.s.	n.s.	n.s.	n.s.
MED23	Mediator complex subunit 23	n.s.	n.s.	n.s.	n.s.	1.86	0.0223
MED8	Mediator complex subunit 8	1.53	0.0098	n.s.	n.s.	1.72	0.0121
METTL23	Methyltransferase like 23	1.52	0.0053	n.s.	n.s.	n.s.	n.s.
MGST3	Microsomal glutathione S-transferase 3	n.s.	n.s.	1.54	0.0023	1.7	0.0005
MIR1183	MicroRNA 1183	-1.6	0.0229	n.s.	n.s.	-1.67	0.0394
MIR1185-1	MicroRNA 1185-1	-1.5	0.0384	n.s.	n.s.	n.s.	n.s.
MIR1185-2	MicroRNA 1185-2	n.s.	n.s.	n.s.	n.s.	-3.46	0.0482
MIR1277	MicroRNA 1277	-1.52	0.007	n.s.	n.s.	n.s.	n.s.
MIR1302-4	MicroRNA 1302-4	n.s.	n.s.	n.s.	n.s.	-1.8	0.0048
MIR193A	MicroRNA 193a	n.s.	n.s.	1.76	0.019	n.s.	n.s.
MIR3668	MicroRNA 3668	n.s.	n.s.	n.s.	n.s.	-1.53	0.0111
MIR384	MicroRNA 384	n.s.	n.s.	n.s.	n.s.	1.79	0.0162
MIR3910-2	MicroRNA 3910-2	n.s.	n.s.	n.s.	n.s.	1.51	0.0469
MIR3975	MicroRNA 3975	n.s.	n.s.	n.s.	n.s.	-1.64	0.0059
MIR4287	MicroRNA 4287	n.s.	n.s.	n.s.	n.s.	-2.6	0.0215
MIR4299	MicroRNA 4299	n.s.	n.s.	n.s.	n.s.	-1.61	0.0496
MIR4434	MicroRNA 4434	n.s.	n.s.	-1.63	0.0206	n.s.	n.s.
MIR4449	MicroRNA 4449	n.s.	n.s.	n.s.	n.s.	-1.77	0.001
MIR4472-1	MicroRNA 4472-1	n.s.	n.s.	n.s.	n.s.	-1.6	0.034
MIR4513	MicroRNA 4513	n.s.	n.s.	n.s.	n.s.	-2.81	0.0086
MIR4521	MicroRNA 4521	-2.09	0.0012	n.s.	n.s.	n.s.	n.s.
MIR4523	MicroRNA 4523	1.56	0.049	n.s.	n.s.	n.s.	n.s.
MIR4682	MicroRNA 4682	n.s.	n.s.	-1.57	0.0009	n.s.	n.s.
MIR4692	MicroRNA 4692	-1.83	0.0282	n.s.	n.s.	n.s.	n.s.
MIR4757	MicroRNA 4757	n.s.	n.s.	n.s.	n.s.	-2.56	0.0167
MIR4801	MicroRNA 4801	n.s.	n.s.	n.s.	n.s.	1.86	0.001
MIR487A	MicroRNA 487a	-1.57	0.0232	n.s.	n.s.	n.s.	n.s.
MIR510	MicroRNA 510	n.s.	n.s.	-1.56	0.038	-1.75	0.0028
MIR514B	MicroRNA 514b	n.s.	n.s.	n.s.	n.s.	-2.14	0.0199
MIR519E	MicroRNA 519e	n.s.	n.s.	n.s.	n.s.	-1.59	0.0288
MIR520B	MicroRNA 520b	n.s.	n.s.	n.s.	n.s.	-1.67	0.0048
MIR539	MicroRNA 539	n.s.	n.s.	1.7	0.0181	n.s.	n.s.
MIR548A2	MicroRNA 548a-2	n.s.	n.s.	n.s.	n.s.	-1.59	0.0047
MIR548A3	MicroRNA 548a-3	n.s.	n.s.	-1.67	0.0454	n.s.	n.s.
MIR548AL	MicroRNA 548al	n.s.	n.s.	2.53	0.0453	n.s.	n.s.
MIR548AN	MicroRNA 548an	-1.54	0.0316	n.s.	n.s.	-1.52	0.0222
MIR548F2	MicroRNA 548f-2	1.74	0.0015	1.7	0.0168	n.s.	n.s.
MIR548F3	MicroRNA 548f-3	n.s.	n.s.	n.s.	n.s.	-1.64	0.0172
MIR548H3	MicroRNA 548h-3	-1.55	0.0437	n.s.	n.s.	n.s.	n.s.
MIR551B	MicroRNA 551b	n.s.	n.s.	n.s.	n.s.	-2.08	0.0287
MIR663A	MicroRNA 663a	-1.71	0.01	n.s.	n.s.	n.s.	n.s.
MIR877	MicroRNA 877	n.s.	n.s.	n.s.	n.s.	-1.58	0.0066
MIR934	MicroRNA 934	n.s.	n.s.	n.s.	n.s.	-2.39	0.0271
MIRLET7C	MicroRNA let-7c	n.s.	n.s.	n.s.	n.s.	-1.58	0.0004
MLLT3	Myeloid/lymphoid or mixed-lineage leukemia; translocated to, 3	n.s.	n.s.	n.s.	n.s.	1.6	3.14E-06
MOSPD1	Motile sperm domain containing 1	n.s.	n.s.	n.s.	n.s.	1.52	0.0256
MPZL2	Myelin protein zero-like 2	n.s.	n.s.	n.s.	n.s.	1.8	0.0295
MRPL34	Mitochondrial ribosomal protein L34	1.52	0.0028	n.s.	n.s.	n.s.	n.s.

MRPL41	Mitochondrial ribosomal protein L41	n.s.	n.s.	1.53	0.0075	n.s.	n.s.
MRPL57	Mitochondrial ribosomal protein L57	n.s.	n.s.	1.63	0.0152	n.s.	n.s.
MRPS10	Mitochondrial ribosomal protein S10	n.s.	n.s.	1.52	2.31E-05	n.s.	n.s.
MT1E	Metallothionein 1E	n.s.	n.s.	2.5	0.0019	n.s.	n.s.
MT1F	Metallothionein 1F	2.24	0.0096	2.79	0.0007	n.s.	n.s.
MT1G	Metallothionein 1G	n.s.	n.s.	2.47	0.0119	n.s.	n.s.
MT1HL1	Metallothionein 1H-like 1	n.s.	n.s.	n.s.	n.s.	-1.5	0.0156
MT1JP	Metallothionein 1J, pseudogene	n.s.	n.s.	1.69	0.024	n.s.	n.s.
MT1L	Metallothionein 1L (gene/pseudogene)	n.s.	n.s.	1.62	0.0001	n.s.	n.s.
MT1X	Metallothionein 1X	n.s.	n.s.	2.39	0.0132	n.s.	n.s.
MT2A	Metallothionein 2A	n.s.	n.s.	1.59	0.0484	n.s.	n.s.
MUT	Methylmalonyl-CoA mutase	1.51	0.0049	n.s.	n.s.	n.s.	n.s.
MYADM	Myeloid-associated differentiation marker	n.s.	n.s.	n.s.	n.s.	1.58	0.0213
MYNN	Myoneurin	1.58	9.38E-06	n.s.	n.s.	n.s.	n.s.
NBPF14; NBPF19; NBPF8	Neuroblastoma breakpoint family, member 14; neuroblastoma breakpoint family, member 19; neuroblastoma breakpoint family, member 8	n.s.	n.s.	n.s.	n.s.	-1.7	0.0282
NBPF9; NBPF10; NBPF14; NOTCH2NL; NBPF19; NBPF8; NBPF25P	Neuroblastoma breakpoint family, member 9; neuroblastoma breakpoint family, member 10; neuroblastoma breakpoint family, member 14; notch 2 N-terminal like; neuroblastoma breakpoint family, member 19; neuroblastoma breakpoint family, member 8; neuroblastoma breakpoint family, member 25, pseudogene	n.s.	n.s.	n.s.	n.s.	-2.1	0.0069
NBPF9; NBPF14; NBPF10; NBPF19; NBPF8; NBPF25P; NBPF20	Neuroblastoma breakpoint family, member 9; neuroblastoma breakpoint family, member 14; neuroblastoma breakpoint family, member 10; neuroblastoma breakpoint family, member 19; neuroblastoma breakpoint family, member 8; neuroblastoma breakpoint family, member 25, pseudogene; neuroblastoma breakpoint family, member 20	n.s.	n.s.	n.s.	n.s.	-2.04	0.0027
NBPF9; NBPF15; NBPF14; NBPF20; NBPF1; NBPF10; NBPF19; NBPF8	Neuroblastoma breakpoint family, member 9; neuroblastoma breakpoint family, member 15; neuroblastoma breakpoint family, member 14; neuroblastoma breakpoint family, member 20; neuroblastoma breakpoint family, member 1; neuroblastoma breakpoint family, member 10; neuroblastoma breakpoint family, member 19; neuroblastoma breakpoint family, member 8	n.s.	n.s.	n.s.	n.s.	-2.13	0.0014
NCBP2-AS2	NCBP2 antisense RNA 2 (head to head)	1.6	0.0047	n.s.	n.s.	n.s.	n.s.
NDUFA12	NADH dehydrogenase (ubiquinone) 1 alpha subcomplex, 12	n.s.	n.s.	2.12	0.0354	n.s.	n.s.
NDUFA4L2	NADH dehydrogenase (ubiquinone) 1 alpha subcomplex, 4-like 2	1.58	0.0133	n.s.	n.s.	1.58	0.0077
NDUFAF7	NADH dehydrogenase (ubiquinone) complex I, assembly factor 7	1.79	0.004	n.s.	n.s.	n.s.	n.s.
NDUFS6	NADH dehydrogenase (ubiquinone) Fe-S protein 6, 13kDa (NADH-coenzyme Q reductase)	n.s.	n.s.	1.7	0.0175	n.s.	n.s.
NFIL3	Nuclear factor, interleukin 3 regulated	1.74	0.0004	n.s.	n.s.	n.s.	n.s.
NFKBIA	Nuclear factor of kappa light polypeptide gene enhancer in B-cells inhibitor, alpha	3.99	5.51E-08	n.s.	n.s.	1.61	0.0015
NFKBIZ	Nuclear factor of kappa light polypeptide gene enhancer in B-cells inhibitor, zeta	1.61	1.56E-05	n.s.	n.s.	n.s.	n.s.
NLK	Nemo-like kinase	n.s.	n.s.	n.s.	n.s.	1.52	0.0087
NPAT	Nuclear protein, ataxia-telangiectasia locus	1.64	0.0151	n.s.	n.s.	1.52	0.0131
NPIPB4; NPIPB3	Nuclear pore complex interacting protein family, member B4; nuclear pore complex interacting protein family, member B3	-1.94	0.0443	n.s.	n.s.	n.s.	n.s.
NR2C2AP	Nuclear receptor 2C2-associated protein	1.54	0.0195	1.57	0.0356	n.s.	n.s.
NRARP	NOTCH-regulated ankyrin repeat protein	n.s.	n.s.	1.51	0.0022	n.s.	n.s.
NRAS	Neuroblastoma RAS viral (v-ras) oncogene homolog	n.s.	n.s.	n.s.	n.s.	1.81	0.0083
NSF; NSFP1	N-ethylmaleimide-sensitive factor; N-ethylmaleimide-sensitive factor pseudogene 1	n.s.	n.s.	n.s.	n.s.	2.07	0.02
NSFP1	N-ethylmaleimide-sensitive factor pseudogene 1	n.s.	n.s.	n.s.	n.s.	1.88	0.0277
NUMB	Numb homolog (Drosophila)	n.s.	n.s.	n.s.	n.s.	1.59	0.0346
OAS1	2-5-oligoadenylate synthetase 1	n.s.	n.s.	n.s.	n.s.	1.77	0.0074

OR11G2	Olfactory receptor, family 11, subfamily G, member 2	-1.53	0.0112	n.s.	n.s.	n.s.	n.s.
OR5H6	Olfactory receptor, family 5, subfamily H, member 6 (gene/pseudogene)	-1.51	0.0178	n.s.	n.s.	n.s.	n.s.
OS9	Osteosarcoma amplified 9, endoplasmic reticulum lectin	n.s.	n.s.	n.s.	n.s.	2.18	0.047
OSBP	Oxysterol binding protein	n.s.	n.s.	n.s.	n.s.	1.52	0.0297
OSBPL9	Oxysterol binding protein-like 9	n.s.	n.s.	n.s.	n.s.	1.94	0.049
OVGP1	Oviductal glycoprotein 1	n.s.	n.s.	n.s.	n.s.	1.6	0.0462
P4HA1	Prolyl 4-hydroxylase, alpha polypeptide I	n.s.	n.s.	n.s.	n.s.	1.6	0.0103
PAFAH1B3	Platelet-activating factor acetylhydrolase 1b, catalytic subunit 3 (29kDa)	n.s.	n.s.	1.54	0.0091	n.s.	n.s.
PAPOLA	Poly(A) polymerase alpha	n.s.	n.s.	n.s.	n.s.	1.67	0.0085
PAPSS2	3-phosphoadenosine 5-phosphosulfate synthase 2	n.s.	n.s.	n.s.	n.s.	1.89	0.0131
PARP4	Poly(ADP-ribose) polymerase family member 4	n.s.	n.s.	n.s.	n.s.	2.12	0.0479
PARS2	Prolyl-tRNA synthetase 2, mitochondrial (putative)	n.s.	n.s.	n.s.	n.s.	1.5	0.0175
PDZK1; PDZK1P1	PDZ domain containing 1; PDZ domain containing 1 pseudogene 1	n.s.	n.s.	n.s.	n.s.	1.55	0.0114
PDZK1P1	PDZ domain containing 1 pseudogene 1	n.s.	n.s.	n.s.	n.s.	1.51	0.0101
PECR	Peroxisomal trans-2-enoyl-CoA reductase	1.64	0.0125	n.s.	n.s.	2.09	0.0204
PEX12	Peroxisomal biogenesis factor 12	n.s.	n.s.	n.s.	n.s.	1.82	0.008
PFDN2	Prefoldin subunit 2	n.s.	n.s.	1.5	0.0259	n.s.	n.s.
PFKP	Phosphofructokinase, platelet	n.s.	n.s.	n.s.	n.s.	1.63	0.0052
PHGR1	Proline/histidine/glycine-rich 1	n.s.	n.s.	1.61	0.0094	n.s.	n.s.
PHIP	Pleckstrin homology domain interacting protein	1.71	0.037	n.s.	n.s.	n.s.	n.s.
PHLDA1	Pleckstrin homology-like domain, family A, member 1	1.51	0.0133	n.s.	n.s.	n.s.	n.s.
PI3	Peptidase inhibitor 3, skin-derived	1.54	0.0016	n.s.	n.s.	n.s.	n.s.
PIGB	Phosphatidylinositol glycan anchor biosynthesis class B	n.s.	n.s.	n.s.	n.s.	1.54	0.0123
PIGBOS1	PIGB opposite strand 1	1.59	0.013	1.73	0.0039	n.s.	n.s.
PIK3C2A	Phosphatidylinositol-4-phosphate 3-kinase, catalytic subunit type 2 alpha	n.s.	n.s.	n.s.	n.s.	1.58	0.0261
PITPNA-AS1	PITPNA antisense RNA 1	n.s.	n.s.	1.54	0.0369	n.s.	n.s.
PLAUR	Plasminogen activator, urokinase receptor	n.s.	n.s.	n.s.	n.s.	1.59	0.0419
PLCB4	Phospholipase C, beta 4	n.s.	n.s.	n.s.	n.s.	1.95	0.0046
PLCL1	Phospholipase C-like 1	n.s.	n.s.	n.s.	n.s.	-1.59	0.0111
PLK2	Polo-like kinase 2	1.58	1.28E-05	n.s.	n.s.	n.s.	n.s.
PLPP6	Phospholipid phosphatase 6	-1.54	0.0152	n.s.	n.s.	-1.61	0.0066
PLSCR4	Phospholipid scramblase 4	n.s.	n.s.	n.s.	n.s.	2.35	0.0038
PNRC2	Proline-rich nuclear receptor coactivator 2	1.5	0.0009	n.s.	n.s.	1.6	2.31E-05
POLD4	Polymerase (DNA-directed), delta 4, accessory subunit	n.s.	n.s.	n.s.	n.s.	1.53	0.0091
POLG2	Polymerase (DNA directed), gamma 2, accessory subunit	1.52	0.0032	n.s.	n.s.	n.s.	n.s.
POLR2I	Polymerase (RNA) II (DNA directed) polypeptide I, 14.5kDa	n.s.	n.s.	1.51	0.0047	n.s.	n.s.
POLR2K	Polymerase (RNA) II (DNA directed) polypeptide K, 7.0kDa	n.s.	n.s.	1.61	0.0079	n.s.	n.s.
PPARG	Peroxisome proliferator-activated receptor gamma	1.55	0.0016	n.s.	n.s.	1.56	0.005
PPIL4	Peptidylprolyl isomerase (cyclophilin)-like 4	1.59	0.0001	n.s.	n.s.	n.s.	n.s.
PPP1R10	Protein phosphatase 1, regulatory subunit 10	-2.41	0.0005	n.s.	n.s.	n.s.	n.s.
PPP1R12A	Protein phosphatase 1, regulatory subunit 12A	n.s.	n.s.	n.s.	n.s.	1.58	0.0007
PPP1R15A	Protein phosphatase 1, regulatory subunit 15A	1.78	0.0011	n.s.	n.s.	n.s.	n.s.
PPWD1	Peptidylprolyl isomerase domain and WD repeat containing 1	1.53	0.0007	n.s.	n.s.	n.s.	n.s.
PREPL	Prolyl endopeptidase-like	n.s.	n.s.	n.s.	n.s.	1.61	0.0029
PRG1	P53-responsive gene 1	-1.56	0.002	n.s.	n.s.	n.s.	n.s.
PROCR	Protein C receptor, endothelial	1.75	0.0119	1.76	0.0327	n.s.	n.s.
PRPF39	Pre-mRNA processing factor 39	1.57	0.0321	n.s.	n.s.	n.s.	n.s.
PRSS22	Protease, serine, 22	n.s.	n.s.	n.s.	n.s.	1.77	0.0432
PSAT1	Phosphoserine aminotransferase 1	n.s.	n.s.	n.s.	n.s.	2.42	0.0412

PSMB6	Polymerase (RNA) II (DNA directed) polypeptide I, 14.5kDa	n.s.	n.s.	1.57	0.0382	n.s.	n.s.
PSME1	Proteasome activator subunit 1	n.s.	n.s.	n.s.	n.s.	1.62	0.046
PTGS2	Prostaglandin-endoperoxide synthase 2 (prostaglandin G/H synthase and cyclooxygenase)	2.86	8.92E-07	n.s.	n.s.	1.53	0.0052
RAB11B-AS1	RAB11B antisense RNA 1	1.66	0.0142	1.7	0.0127	n.s.	n.s.
RAB14	RAB14, member RAS oncogene family	n.s.	n.s.	n.s.	n.s.	1.65	0.0121
RAB20	RAB20, member RAS oncogene family	1.58	0.0038	n.s.	n.s.	n.s.	n.s.
RAB27A	RAB27A, member RAS oncogene family	n.s.	n.s.	n.s.	n.s.	1.63	0.0214
RAB27B	RAB27B, member RAS oncogene family	n.s.	n.s.	n.s.	n.s.	1.58	0.0013
RAB30	RAB30, member RAS oncogene family	1.51	0.006	n.s.	n.s.	n.s.	n.s.
RAB3B	RAB3B, member RAS oncogene family	n.s.	n.s.	n.s.	n.s.	1.81	0.0156
RASA1	RAS p21 protein activator (GTPase activating protein) 1	n.s.	n.s.	n.s.	n.s.	1.63	0.0193
RBBP9	Retinoblastoma binding protein 9	n.s.	n.s.	1.53	0.0351	2	0.0126
RHOB	Ras homolog family member B	1.54	0.0053	n.s.	n.s.	n.s.	n.s.
RHOU	Ras homolog family member U	1.77	0.0002	n.s.	n.s.	n.s.	n.s.
RIPK2	Receptor-interacting serine-threonine kinase 2	1.54	0.0216	n.s.	n.s.	n.s.	n.s.
RNA5S1	RNA, 5S ribosomal 1	-3.31	0.0008	n.s.	n.s.	-2.19	0.011
RNA5S10	RNA, 5S ribosomal 10	-3.31	0.0008	n.s.	n.s.	-2.19	0.011
RNA5S11	RNA, 5S ribosomal 11	-3.31	0.0008	n.s.	n.s.	-2.19	0.011
RNA5S12	RNA, 5S ribosomal 12	-3.31	0.0008	n.s.	n.s.	-2.19	0.011
RNA5S13	RNA, 5S ribosomal 13	-3.31	0.0008	n.s.	n.s.	-2.19	0.011
RNA5S14	RNA, 5S ribosomal 14	-3.31	0.0008	n.s.	n.s.	-2.19	0.011
RNA5S15	RNA, 5S ribosomal 15	-3.31	0.0008	n.s.	n.s.	-2.19	0.011
RNA5S16	RNA, 5S ribosomal 16	-3.31	0.0008	n.s.	n.s.	-2.19	0.011
RNA5S17	RNA, 5S ribosomal 17	-3.31	0.0008	n.s.	n.s.	-2.19	0.011
RNA5S2	RNA, 5S ribosomal 2	-3.31	0.0008	n.s.	n.s.	-2.19	0.011
RNA5S3	RNA, 5S ribosomal 3	-3.31	0.0008	n.s.	n.s.	-2.19	0.011
RNA5S4	RNA, 5S ribosomal 4	-3.31	0.0008	n.s.	n.s.	-2.19	0.011
RNA5S5	RNA, 5S ribosomal 5	-3.31	0.0008	n.s.	n.s.	-2.19	0.011
RNA5S6	RNA, 5S ribosomal 6	-3.31	0.0008	n.s.	n.s.	-2.19	0.011
RNA5S7	RNA, 5S ribosomal 7	-3.31	0.0008	n.s.	n.s.	-2.19	0.011
RNA5S8	RNA, 5S ribosomal 8	-3.31	0.0008	n.s.	n.s.	-2.19	0.011
RNA5S9	RNA, 5S ribosomal 9	-4.49	0.0019	n.s.	n.s.	-3.65	0.015
RNA5SP348	RNA, 5S ribosomal pseudogene 348	-5.55	0.0412	n.s.	n.s.	n.s.	n.s.
RNA5SP357	RNA, 5S ribosomal pseudogene 357	-1.57	0.0246	n.s.	n.s.	n.s.	n.s.
RNA5SP381	RNA, 5S ribosomal pseudogene 381	-2.38	0.0007	n.s.	n.s.	n.s.	n.s.
RNA5SP386	RNA, 5S ribosomal pseudogene 386	-1.58	0.0054	-1.52	0.0271	n.s.	n.s.
RNA5SP439	RNA, 5S ribosomal pseudogene 439	-1.62	0.0189	n.s.	n.s.	n.s.	n.s.
RNA5SP440	RNA, 5S ribosomal pseudogene 440	-3.16	0.0083	n.s.	n.s.	-3.56	0.0246
RNA5SP506	RNA, 5S ribosomal pseudogene 506	-2.67	0.0023	n.s.	n.s.	-2.01	0.0454
RND1	Rho family GTPase 1	2.01	2.17E-06	n.s.	n.s.	n.s.	n.s.
RND3	Rho family GTPase 3	1.79	0.0004	n.s.	n.s.	n.s.	n.s.
RNF128	Ring finger protein 128, E3 ubiquitin protein ligase	n.s.	n.s.	n.s.	n.s.	1.72	0.0099
RNF39	Ring finger protein 39	n.s.	n.s.	n.s.	n.s.	1.75	0.0489
RNU11	RNA, U11 small nuclear	n.s.	n.s.	n.s.	n.s.	-6.46	0.0388
RNU1-10P	RNA, U1 small nuclear 10, pseudogene	n.s.	n.s.	n.s.	n.s.	-1.6	0.0488
RNU6-76P	RNA, U6 small nuclear 76, pseudogene	n.s.	n.s.	n.s.	n.s.	-1.9	0.0487
RNVU1-1	RNA, variant U1 small nuclear 1	n.s.	n.s.	n.s.	n.s.	-1.6	0.0488
ROCK1	Rho-associated, coiled-coil containing protein kinase 1	n.s.	n.s.	n.s.	n.s.	1.63	0.0098
ROCK1P1	Rho-associated, coiled-coil containing protein kinase 1 pseudogene 1	n.s.	n.s.	n.s.	n.s.	1.52	0.005
RP11-436H11.3	Long non-coding RNA	n.s.	n.s.	n.s.	n.s.	-1.51	0.0065

RP11-90P5.7	Long non-coding RNA	n.s.	n.s.	n.s.	n.s.	-4.31	0.0482
RP4-655J12.5	Long non-coding RNA	-1.63	0.0037	n.s.	n.s.	n.s.	n.s.
RPF1	Ribosome production factor 1 homolog	n.s.	n.s.	1.72	0.0474	n.s.	n.s.
RPF2	Ribosome production factor 2 homolog	n.s.	n.s.	n.s.	n.s.	1.75	0.0041
RPL10A	Ribosomal protein L10a	1.55	0.0394	1.54	0.0197	n.s.	n.s.
RPL36	Ribosomal protein L36	n.s.	n.s.	1.67	0.0094	n.s.	n.s.
RPL41	Ribosomal protein L41	n.s.	n.s.	1.53	0.0366	n.s.	n.s.
RPS18	Ribosomal protein S18	1.81	0.0007	1.6	0.0053	n.s.	n.s.
RPS19	Ribosomal protein S19	n.s.	n.s.	1.67	0.0341	n.s.	n.s.
RPS28	Ribosomal protein S28	1.6	1.13E-06	1.57	1.46E-06	1.62	1.95E-06
RPS29	Ribosomal protein S29	2.12	0.0087	2.43	0.0013	n.s.	n.s.
RRAGA	Ras-related GTP binding A	1.64	0.0333	2.21	0.0002	1.79	0.0007
RSPRY1	Ring finger and SPRY domain containing 1	n.s.	n.s.	n.s.	n.s.	1.63	0.0192
RSRC2	Arginine/serine-rich coiled-coil 2	n.s.	n.s.	n.s.	n.s.	1.53	0.0005
RYBP	RING1 and YY1 binding protein	1.7	0.0172	n.s.	n.s.	1.75	0.0005
SAMD9	Sterile alpha motif domain containing 9	1.52	0.0282	n.s.	n.s.	1.92	0.0006
SCARNA12	Small Cajal body-specific RNA 12	n.s.	n.s.	n.s.	n.s.	-2.49	0.032
SCGB2A1	Secretoglobin, family 2A, member 1	n.s.	n.s.	1.71	0.0343	n.s.	n.s.
SCIN	Scinderin	n.s.	n.s.	n.s.	n.s.	1.73	0.0065
SCUBE1	Signal peptide, CUB domain, EGF-like 1	n.s.	n.s.	n.s.	n.s.	-1.63	0.0258
SDC4	Syndecan 4	2.08	0.0341	n.s.	n.s.	n.s.	n.s.
SEC24A	SEC24 homolog A, COPII coat complex component	n.s.	n.s.	n.s.	n.s.	1.61	0.0342
SECISBP2L	SECIS binding protein 2-like	n.s.	n.s.	n.s.	n.s.	1.62	0.0113
SENP7	SUMO1/sentrin specific peptidase 7	n.s.	n.s.	n.s.	n.s.	1.54	0.0263
SERINC1	Serine incorporator 1	n.s.	n.s.	n.s.	n.s.	1.72	0.0473
SERTAD2	SERTA domain containing 2	1.7	0.0144	n.s.	n.s.	n.s.	n.s.
SERTAD3	SERTA domain containing 3	1.87	7.48E-05	1.55	0.0106	n.s.	n.s.
SGMS1-AS1	SGMS1 antisense RNA 1	n.s.	n.s.	n.s.	n.s.	1.67	0.0245
SGMS2	Sphingomyelin synthase 2	n.s.	n.s.	n.s.	n.s.	1.5	0.0025
SGMS2	Sphingomyelin synthase 2	n.s.	n.s.	n.s.	n.s.	-1.75	0.0037
SGPP2	Sphingosine-1-phosphate phosphatase 2	n.s.	n.s.	n.s.	n.s.	1.6	0.0119
SH3BGRL2	SH3 domain binding glutamate-rich protein like 2	n.s.	n.s.	n.s.	n.s.	1.68	0.0017
SH3PXD2A-AS1	SH3PXD2A antisense RNA 1	n.s.	n.s.	n.s.	n.s.	1.78	0.0248
SHH	Sonic hedgehog	n.s.	n.s.	n.s.	n.s.	1.8	0.0083
SI	Sucrase-isomaltase (alpha-glucosidase)	1.7	0.0447	n.s.	n.s.	n.s.	n.s.
SKP2	S-phase kinase-associated protein 2, E3 ubiquitin protein ligase	n.s.	n.s.	n.s.	n.s.	1.75	0.0196
SLC10A7	Solute carrier family 10, member 7	n.s.	n.s.	n.s.	n.s.	1.69	0.0418
SLC25A16	Solute carrier family 25 (mitochondrial carrier), member 16	1.51	0.0119	1.53	0.0229	n.s.	n.s.
SLC30A1	Solute carrier family 30 (zinc transporter), member 1	1.91	0.0048	n.s.	n.s.	n.s.	n.s.
SLC31A1	Solute carrier family 31 (copper transporter), member 1	n.s.	n.s.	n.s.	n.s.	2.32	0.0174
SLC35A5	Solute carrier family 35, member A5	n.s.	n.s.	n.s.	n.s.	1.6	0.007
SLC38A2	Solute carrier family 38, member 2	n.s.	n.s.	n.s.	n.s.	2.12	0.0195
SLC3A2	Solute carrier family 3 (amino acid transporter heavy chain), member 2	2.37	0.0047	n.s.	n.s.	n.s.	n.s.
SLC7A5	Solute carrier family 7 (amino acid transporter light chain, L system), member 5	n.s.	n.s.	n.s.	n.s.	2.88	0.0493
SMARCA1	SWI/SNF related, matrix associated, actin dependent regulator of chromatin, subfamily a, member 1	n.s.	n.s.	n.s.	n.s.	1.54	0.0079
SMDT1	Single-pass membrane protein with aspartate-rich tail 1	2.08	0.0105	2.22	0.003	2.46	0.0056
SMG1P2	SMG1 pseudogene 2	1.71	0.0384	n.s.	n.s.	n.s.	n.s.
SMOX	Spermine oxidase	1.56	0.0017	n.s.	n.s.	n.s.	n.s.
SNHG17	Small nucleolar RNA host gene 17	1.54	0.0017	n.s.	n.s.	n.s.	n.s.

SNORA2A	Small nucleolar RNA, H/ACA box 2A	n.s.	n.s.	n.s.	n.s.	-4.65	0.0203
SNORA38B	Small nucleolar RNA, H/ACA box 38B	n.s.	n.s.	1.85	0.0171	n.s.	n.s.
SNORA46	Small nucleolar RNA, H/ACA box 46	n.s.	n.s.	n.s.	n.s.	-2.56	0.023
SNORA55	Small nucleolar RNA, H/ACA box 55	n.s.	n.s.	n.s.	n.s.	-1.62	0.0274
SNORA74B	Small nucleolar RNA, H/ACA box 74B	n.s.	n.s.	n.s.	n.s.	-1.83	0.0207
SNORA79	Small nucleolar RNA, H/ACA box 79	n.s.	n.s.	n.s.	n.s.	-2.78	0.0499
SNORD101; SNORD100; SNORA33	Small nucleolar RNA, C/D box 101; small nucleolar RNA, C/D box 100; small nucleolar RNA, H/ACA box 33	n.s.	n.s.	n.s.	n.s.	-2.35	0.0344
SNORD104	Small nucleolar RNA, C/D box 104	-3.74	0.002	-3.39	0.0097	-4.89	0.0002
SNORD105B	Small nucleolar RNA, C/D box 105B	-2.08	0.0018	n.s.	n.s.	-1.87	0.0139
SNORD111B	Small nucleolar RNA, C/D box 111B	n.s.	n.s.	n.s.	n.s.	-3.4	0.0424
SNORD113-1	Small nucleolar RNA, C/D box 113-1	1.59	0.0149	1.77	0.0034	n.s.	n.s.
SNORD115-40	Small nucleolar RNA, C/D box 115-40	-1.63	0.0327	n.s.	n.s.	n.s.	n.s.
SNORD115-45	Small nucleolar RNA, C/D box 115-45	-1.94	0.0101	n.s.	n.s.	n.s.	n.s.
SNORD115-48	Small nucleolar RNA, C/D box 115-48	n.s.	n.s.	n.s.	n.s.	-2.49	0.0002
SNORD126	Small nucleolar RNA, C/D box 126	n.s.	n.s.	n.s.	n.s.	-6.82	0.045
SNORD14E	Small nucleolar RNA, C/D box 14E	n.s.	n.s.	n.s.	n.s.	-4.67	0.008
SNORD41	Small nucleolar RNA, C/D box 41	-3.52	0.0055	n.s.	n.s.	n.s.	n.s.
SNORD53	Small nucleolar RNA, C/D box 53	n.s.	n.s.	n.s.	n.s.	-2.85	0.0416
SNORD56B	Small nucleolar RNA, C/D box 56B	n.s.	n.s.	n.s.	n.s.	-1.63	0.0322
SNORD58A	Small nucleolar RNA, C/D box 58A	-1.95	0.0455	n.s.	n.s.	n.s.	n.s.
SNORD60	Small nucleolar RNA, C/D box 60	-2.3	0.0287	n.s.	n.s.	-4.24	0.0281
SNORD62A; SNORD62B	Small nucleolar RNA, C/D box 62A; small nucleolar RNA, C/D box 62B	n.s.	n.s.	n.s.	n.s.	-3.3	0.0254
SNORD62B; SNORD62A	Small nucleolar RNA, C/D box 62B; small nucleolar RNA, C/D box 62A	n.s.	n.s.	n.s.	n.s.	-3.3	0.0254
SNORD66	Small nucleolar RNA, C/D box 66	n.s.	n.s.	n.s.	n.s.	-2.79	0.0273
SNORD71	Small nucleolar RNA, C/D box 71	-2.85	0.0043	n.s.	n.s.	-2.78	0.0086
SNORD93	Small nucleolar RNA, C/D box 93	n.s.	n.s.	n.s.	n.s.	-3.31	0.0014
SNORD99	Small nucleolar RNA, C/D box 99	-1.67	0.0358	n.s.	n.s.	n.s.	n.s.
SNTB1	Syntrophin, beta 1 (dystrophin-associated protein A1, 59kDa, basic component 1)	n.s.	n.s.	n.s.	n.s.	1.82	0.0234
SNW1	SNW domain containing 1	n.s.	n.s.	n.s.	n.s.	1.56	0.0016
SNX27	Sorting nexin family member 27	-1.54	0.0049	n.s.	n.s.	n.s.	n.s.
SPATS2L	Spermatogenesis associated, serine-rich 2-like	n.s.	n.s.	n.s.	n.s.	1.51	0.0244
SPG11	Spastic paraplegia 11 (autosomal recessive)	n.s.	n.s.	n.s.	n.s.	1.6	0.0427
SPOP	Speckle-type POZ protein	1.56	0.0478	n.s.	n.s.	2.43	0.0475
SPRR1A	Small proline-rich protein 1A	n.s.	n.s.	n.s.	n.s.	1.88	0.0363
SPRR1B	Small proline-rich protein 1B	n.s.	n.s.	n.s.	n.s.	2.4	0.0133
SPRR3	Small proline-rich protein 3	n.s.	n.s.	n.s.	n.s.	1.82	0.006
SRD5A3	Steroid 5 alpha-reductase 3	n.s.	n.s.	n.s.	n.s.	1.74	0.0362
SRI	Sorcin	n.s.	n.s.	1.6	0.0166	n.s.	n.s.
SRSF10	Serine/arginine-rich splicing factor 10	1.63	0.0046	n.s.	n.s.	n.s.	n.s.
SRSF7	Serine/arginine-rich splicing factor 7	1.81	0.0002	n.s.	n.s.	n.s.	n.s.
SSBP2	Single-stranded DNA binding protein 2	n.s.	n.s.	n.s.	n.s.	1.63	0.0028
ST13P4	Suppression of tumorigenicity 13 (colon carcinoma) (Hsp70 interacting protein) pseudogene 4	n.s.	n.s.	n.s.	n.s.	1.6	0.0029
ST6GALNAC2	ST6 (alpha-N-acetyl-neuraminyl-2,3-beta-galactosyl-1,3)-N-acetylgalactosaminide alpha-2,6-sialyltransferase 2	n.s.	n.s.	n.s.	n.s.	1.59	0.0129
STK17A	Serine/threonine kinase 17a	1.59	6.55E-05	n.s.	n.s.	n.s.	n.s.
STK17B	Serine/threonine kinase 17b	1.55	0.0059	n.s.	n.s.	n.s.	n.s.
STX12	Syntaxin 12	n.s.	n.s.	n.s.	n.s.	1.57	0.0029
SUCLG2	Succinate-CoA ligase, GDP-forming, beta subunit	n.s.	n.s.	n.s.	n.s.	1.56	0.0061
SUPT4H1	SPT4 homolog, DSIF elongation factor subunit	n.s.	n.s.	1.5	0.0007	n.s.	n.s.
SWAP70	SWAP switching B-cell complex 70kDa subunit	n.s.	n.s.	n.s.	n.s.	1.81	0.0259

SYTL5	Synaptotagmin-like 5	n.s.	n.s.	n.s.	n.s.	2.01	0.0187
SYVN1	Synovial apoptosis inhibitor 1, synoviolin	n.s.	n.s.	n.s.	n.s.	1.98	0.0453
TANGO6	Transport and golgi organization 6 homolog	1.84	0.0008	n.s.	n.s.	n.s.	n.s.
TBC1D20	TBC1 domain family, member 20	n.s.	n.s.	n.s.	n.s.	1.68	0.0377
TBC1D8B	TBC1 domain family, member 8B (with GRAM domain)	n.s.	n.s.	n.s.	n.s.	1.6	0.0158
TBCK	TBC1 domain containing kinase	n.s.	n.s.	n.s.	n.s.	1.81	0.0262
TCEAL8	Transcription elongation factor A (SII)-like 8	n.s.	n.s.	n.s.	n.s.	1.5	0.0164
TFF2	Trefoil factor 2	2.18	0.0134	2.38	0.0029	2.26	0.0003
TIFA	TRAF-interacting protein with forkhead-associated domain	1.57	0.0088	n.s.	n.s.	n.s.	n.s.
TIGD2	Tigger transposable element derived 2	2.19	0.0044	n.s.	n.s.	1.67	0.0281
TIMM8B	Translocase of inner mitochondrial membrane 8 homolog B (yeast)	n.s.	n.s.	1.53	0.032	n.s.	n.s.
TIMP1	TIMP metallopeptidase inhibitor 1	n.s.	n.s.	n.s.	n.s.	1.74	0.0476
TIPARP	TCDD-inducible poly(ADP-ribose) polymerase	1.72	0.0002	n.s.	n.s.	n.s.	n.s.
TLCD1	TLC domain containing 1	1.56	0.0027	1.62	0.0023	n.s.	n.s.
TLR6	Toll-like receptor 6	n.s.	n.s.	n.s.	n.s.	1.72	0.005
TM4SF5	Transmembrane 4 L six family member 5	2.27	0.0002	2	0.0081	2.57	0.00002
TMEM101	Transmembrane protein 101	1.51	0.0052	n.s.	n.s.	n.s.	n.s.
TMEM185A	Transmembrane protein 185A	n.s.	n.s.	n.s.	n.s.	2.31	0.0454
TMEM205	Transmembrane protein 205	n.s.	n.s.	1.52	0.002	n.s.	n.s.
TMEM208	Transmembrane protein 208	n.s.	n.s.	1.6	0.0083	n.s.	n.s.
TMEM238	Transmembrane protein 238	n.s.	n.s.	n.s.	n.s.	1.78	0.0067
TMEM50A	Transmembrane protein 50A	n.s.	n.s.	n.s.	n.s.	1.71	0.0009
TMEM57	Transmembrane protein 57	n.s.	n.s.	n.s.	n.s.	1.7	0.0073
TMEM69	Transmembrane protein 69	n.s.	n.s.	n.s.	n.s.	1.58	0.0043
TMEM87A	Transmembrane protein 87A	n.s.	n.s.	n.s.	n.s.	1.74	0.0423
TNFAIP2	Tumor necrosis factor, alpha-induced protein 2	1.5	0.0011	n.s.	n.s.	1.55	0.0114
TNFAIP3	Tumor necrosis factor, alpha-induced protein 3	2.95	3.58E-10	n.s.	n.s.	n.s.	n.s.
TNFSF10	Tumor necrosis factor (ligand) superfamily, member 10	n.s.	n.s.	n.s.	n.s.	1.85	0.0012
TNIK	TRAF2 and NCK interacting kinase	n.s.	n.s.	n.s.	n.s.	1.57	0.0472
TOR1B	Torsin family 1, member B (torsin B)	n.s.	n.s.	n.s.	n.s.	1.51	0.0094
TPCN1	Two pore segment channel 1	n.s.	n.s.	n.s.	n.s.	1.88	0.0428
TPMT	Thiopurine S-methyltransferase	1.57	0.0006	n.s.	n.s.	n.s.	n.s.
TPRG1L	Tumor protein p63 regulated 1-like	n.s.	n.s.	n.s.	n.s.	1.8	0.0404
TRAPPC6B	Trafficking protein particle complex 6B	1.53	0.0051	1.5	0.0091	1.67	0.0003
TRIM31	Tripartite motif containing 31	1.74	0.0055	n.s.	n.s.	1.93	0.011
TTC39A	Tetratricopeptide repeat domain 39A	n.s.	n.s.	n.s.	n.s.	1.65	0.0248
TUG1	Taurine up-regulated 1 (non-protein coding)	n.s.	n.s.	n.s.	n.s.	1.7	0.0474
TXNDC12	Thioredoxin domain containing 12 (endoplasmic reticulum)	1.64	0.0459	n.s.	n.s.	n.s.	n.s.
UBQLN2	Ubiquilin 2	n.s.	n.s.	n.s.	n.s.	1.61	9.81E-05
UFL1	UFM1-specific ligase 1	n.s.	n.s.	n.s.	n.s.	1.61	0.0115
UFSP1	UFM1-specific peptidase 1 (inactive)	1.6	0.0042	n.s.	n.s.	n.s.	n.s.
UQCRHL	Ubiquinol-cytochrome c reductase hinge protein like	n.s.	n.s.	1.73	0.008	1.52	0.0204
USP17L9P; USP17L11; USP17L20; USP17L22; USP17L17	Ubiquitin specific peptidase 17-like family member 9, pseudogene; ubiquitin specific peptidase 17-like family member 11; ubiquitin specific peptidase 17-like family member 20; ubiquitin specific peptidase 17-like family member 22; ubiquitin specific peptidase 17-like family member 17	-1.66	0.0162	n.s.	n.s.	n.s.	n.s.
USP37	Ubiquitin specific peptidase 37	1.61	0.0408	n.s.	n.s.	n.s.	n.s.
USP53	Ubiquitin specific peptidase 53	n.s.	n.s.	n.s.	n.s.	1.56	0.0077
VAMP3	Vesicle associated membrane protein 3	n.s.	n.s.	n.s.	n.s.	1.6	0.0071
VEZF1	Vascular endothelial zinc finger 1	1.63	0.0308	n.s.	n.s.	1.64	0.0113

VPS35	VPS35 retromer complex component	n.s.	n.s.	n.s.	n.s.	2.04	0.0387
VPS52	Vacuolar protein sorting 52 homolog (S. cerevisiae)	n.s.	n.s.	n.s.	n.s.	1.59	0.0109
VTRNA1-2	Vault RNA 1-2	n.s.	n.s.	n.s.	n.s.	-1.52	0.0319
VTRNA1-3	Vault RNA 1-3	n.s.	n.s.	n.s.	n.s.	-1.82	0.0231
WASF2	WAS protein family, member 2	n.s.	n.s.	n.s.	n.s.	1.69	0.0138
WBP11	WW domain binding protein 11	n.s.	n.s.	n.s.	n.s.	1.67	0.0333
WDR75	WD repeat domain 75	1.62	0.0037	n.s.	n.s.	n.s.	n.s.
XXYLT1-AS2	XXYLT1 antisense RNA 2	n.s.	n.s.	1.58	0.0012	n.s.	n.s.
YWHAB	Tyrosine 3-monooxygenase/tryptophan 5-monooxygenase activation protein, beta	n.s.	n.s.	n.s.	n.s.	1.59	0.0015
ZBTB2	Zinc finger and BTB domain containing 2	1.51	0.0257	n.s.	n.s.	n.s.	n.s.
ZC2HC1A	Zinc finger, C2HC-type containing 1A	1.56	0.0002	n.s.	n.s.	n.s.	n.s.
ZDHHC7	Zinc finger, DHHC-type containing 7	n.s.	n.s.	n.s.	n.s.	1.6	0.0232
ZMYM5	Zinc finger, MYM-type 5	1.6	0.0001	n.s.	n.s.	n.s.	n.s.
ZMYND8	Zinc finger, MYND-type containing 8	n.s.	n.s.	n.s.	n.s.	1.88	0.0188
ZNF143	Zinc finger protein 143	1.53	0.0033	n.s.	n.s.	n.s.	n.s.
ZNF146	Zinc finger protein 146	n.s.	n.s.	n.s.	n.s.	2.14	0.0322
ZNF17	Zinc finger protein 17	1.61	0.0432	n.s.	n.s.	n.s.	n.s.
ZNF33A; ZNF25	Zinc finger protein 33A; zinc finger protein 25	n.s.	n.s.	n.s.	n.s.	1.56	0.0466
ZNF416	Zinc finger protein 416	1.53	0.014	n.s.	n.s.	n.s.	n.s.
ZNF419	Zinc finger protein 419	1.56	0.0004	n.s.	n.s.	n.s.	n.s.
ZNF543	Zinc finger protein 543	1.65	0.0017	n.s.	n.s.	n.s.	n.s.
ZNF585A	Zinc finger protein 585A	n.s.	n.s.	n.s.	n.s.	1.65	0.0321
ZNF623	Zinc finger protein 623	n.s.	n.s.	n.s.	n.s.	1.57	0.0073
ZNF654	Zinc finger protein 654	n.s.	n.s.	n.s.	n.s.	1.66	0.0341
ZNF805	Zinc finger protein 805	1.54	0.035	n.s.	n.s.	n.s.	n.s.

[a] n.s.: not significant.

Table S2. List of proteins identified in *E. gallinarum* MRx0518 late log culture supernatants as identified by nanoLC-MS/MS. This table lists the top 50 hits with the highest PSM values, identified in at least two biological replicates.

Annotation	PSM value [a]		RAST functional category [b]	PSORTb localization (score) [c]	MW (kDa) [d]	pI [d]
	Average	SD				
Enolase (EC 4.2.1.11)	76.00	4.36	Carbohydrates	C (9.97)	78.6	4.98
Oligopeptide ABC transporter, periplasmic oligopeptide-binding protein OppA (TC 3.A.1.5.1)	71.67	6.11	Regulation and cell signaling	CM/CW/E (3.33)	71.7	4.59
Flagellin protein FlaA	71.33	3.79	Motility and chemotaxis	n.d. (2.5)	57.4	4.93
Predicted nucleoside ABC transporter, substrate-binding component	66.33	4.51	Carbohydrates	CM/CW/E (3.33)	33.9	5.06
Lipoteichoic acid synthase LtaS Type IIc	54.00	2.65	Cell wall and capsule	CM (10)	31.3	5.49
NAD-dependent glyceraldehyde-3-phosphate dehydrogenase (EC 1.2.1.12)	50.67	3.21	Stress response	C (9.97)	37.5	5.91
Translation elongation factor Tu	47.67	3.51	Virulence, disease and defense	C (9.97)	36.2	6.29
Heat shock protein 60 family chaperone GroEL	47.33	2.08	Protein metabolism	C (9.97)	28.2	6.07
Pyruvate formate-lyase (EC 2.3.1.54)	47.33	3.21	Carbohydrates	C (9.97)	37.9	6.15
Non-specific DNA-binding protein Dps / Iron-binding ferritin-like antioxidant protein / Ferroxidase (EC 1.16.3.1)	46.33	10.07	Stress response	C (9.97)	16.0	7.18
Alcohol dehydrogenase (EC 1.1.1.1); Acetaldehyde dehydrogenase (EC 1.2.1.10)	46.00	2.65	Carbohydrates	C (9.97)	26.0	7.93
Non-specific DNA-binding protein Dps / Iron-binding ferritin-like antioxidant protein / Ferroxidase (EC 1.16.3.1)	46.00	9.17	Stress response	C (9.97)	43.2	5.67
Pyruvate kinase (EC 2.7.1.40)	46.00	2.65	Carbohydrates	C (7.5)	45.8	4.92
Chaperone protein DnaK	45.33	3.79	Protein metabolism	C (9.97)	24.6	5.41
Translation elongation factor G	38.00	1.00	Virulence, disease and defense	C (9.97)	34.3	5.85
Glycerol-3-phosphate ABC transporter, periplasmic glycerol-3-phosphate-binding protein (TC 3.A.1.1.3)	35.00	4.00	Carbohydrates	CM/CW/E (3.33)	52.8	5.03
Manganese ABC transporter, periplasmic-binding protein SitA	34.67	2.52	n.d.	CM (10)	37.9	4.84
Phosphoglycerate kinase (EC 2.7.2.3)	34.67	0.58	Carbohydrates	C (9.97)	38.5	4.98
Hypothetical protein	33.67	2.08	n.d.	E (9.73)	33.8	4.59
Hypothetical protein	33.00	1.73	n.d.	E (9.73)	37.1	4.73
Fumarate reductase flavoprotein subunit (EC 1.3.99.1)	32.67	1.53	Respiration	CM (9.51)	27.7	8.06
Glucose-6-phosphate isomerase (EC 5.3.1.9)	31.67	2.52	Carbohydrates	C (9.97)	40.2	4.49
Cell division protein FtsI [Peptidoglycan synthetase] (EC 2.4.1.129)	30.00	1.00	RNA metabolism	CM (9.51)	59.3	4.82
Putative E protein	29.67	3.51	n.d.	CM/CW/E (3.33)	32.1	4.84
Acetylornithine deacetylase/Succinyl-diaminopimelate desuccinylase and related deacylases	26.67	1.15	n.d.	C (7.5)	67.3	9.32
Phosphoenolpyruvate-protein phosphotransferase of PTS system (EC 2.7.3.9)	25.33	1.53	Carbohydrates	C (9.97)	69.5	5.39
Translation elongation factor Ts	25.33	1.15	Protein metabolism	C (9.97)	24.6	5.55
Cell division protein FtsI [Peptidoglycan synthetase] (EC 2.4.1.129)	24.67	0.58	RNA metabolism	CM (9.51)	43.6	4.97
Fructose-bisphosphate aldolase class II (EC 4.1.2.13)	24.33	1.15	Carbohydrates	C (7.5)	32.3	6.70
DNA-directed RNA polymerase beta' subunit (EC 2.7.7.6)	24.00	0.00	Virulence, disease and defense	C (9.97)	51.9	4.77
Phosphoglycerate mutase (EC 5.4.2.1)	23.67	0.58	Miscellaneous	C (7.5)	8.6	4.15
6-phosphogluconate dehydrogenase, decarboxylating (EC 1.1.1.44)	23.33	0.58	Carbohydrates	C (7.5)	74.8	5.10
Glutamyl aminopeptidase (EC 3.4.11.7); Deblocking aminopeptidase	22.67	1.53	n.d.	C (7.5)	18.8	5.10
CW-binding protein	22.33	1.53	n.d.	CM/CW/E (3.33)	23.8	5.11
Lipoprotein, putative	22.00	1.00	n.d.	CM/CW/E (3.33)	47.7	5.58

Transketolase (EC 2.2.1.1)	21.33	1.53	Carbohydrates	n.d. (2.5)	21.6	5.22
DNA-directed RNA polymerase beta subunit (EC 2.7.7.6)	21.00	3.46	Virulence, disease and defense	C (9.97)	21.1	4.41
Serine protease, DegP/HtrA, do-like (EC 3.4.21.-)	21.00	2.00	n.d.	n.d. (2.5)	97.2	5.14
LSU ribosomal protein L5p (L11e)	19.67	1.53	Protein metabolism	C (7.5)	35.0	5.38
Membrane-bound lytic murein transglycosylase D precursor (EC 3.2.1.-)	19.67	0.58	Cell wall and capsule	E (9.73)	94.1	6.19
Arginyl-tRNA synthetase (EC 6.1.1.19)	19.00	1.73	Protein metabolism	C (9.97)	20.8	4.81
L-lactate dehydrogenase (EC 1.1.1.27)	18.67	3.06	Carbohydrates	C (9.97)	57.3	4.93
Hypothetical protein	18.33	1.53	n.d.	E (9.98)	49.9	4.96
Glutamine synthetase type I (EC 6.3.1.2)	18.33	1.53	Cell wall and capsule	C (9.97)	44.8	4.88
Phosphoglucomutase (EC 5.4.2.2)	18.00	0.00	n.d.	C (7.5)	38.7	4.68
Threonine synthase (EC 4.2.3.1)	17.67	0.58	Amino Acids and Derivatives	C (7.5)	46.5	4.93
CTP synthase (EC 6.3.4.2)	17.33	1.15	Nucleosides and nucleotides	C (7.5)	63.8	5.35
Hypothetical protein	17.33	1.53	n.d.	C (7.5)	72.5	5.81
Pheromone cAD1 precursor lipoprotein Cad	16.67	2.89	Regulation and cell signaling	CM/CW/E (3.33)	49.4	5.26
Cell division trigger factor (EC 5.2.1.8)	16.00	1.73	n.d.	C (7.5)	45.9	5.43

[a] Average peptide spectrum match values from at least 2 biological replicates and corresponding standard deviation values.

[b] Subsystem category distribution as annotated by RAST (1, 2). n.d. not determined.

[c] Cellular localization as predicted using PSORTb v3.0 (3). C: cytoplasmic, CM: cytoplasmic membrane, CW: cell wall, E: extracellular, n.d.: not determined.

[d] Parameters calculated by Proteome Discoverer (Thermo Scientific, Waltham, MA, USA). MW: molecular weight, pI: isoelectric point.

References

1. Aziz RK, Bartels D, Best AA, DeJongh M, Disz T, Edwards RA, Formsma K, Gerdes S, Glass EM, Kubal M, Meyer F, Olsen GJ, Olson R, Osterman AL, Overbeek RA, McNeil LK, Paarmann D, Paczian T, Parrello B, Pusch GD, Reich C, Stevens R, Vassieva O, Vonstein V, Wilke A, Zagnitko O. 2008. The RAST Server: rapid annotations using subsystems technology. BMC Genomics 9:75.
2. Overbeek R, Olson R, Pusch GD, Olsen GJ, Davis JJ, Disz T, Edwards RA, Gerdes S, Parrello B, Shukla M, Vonstein V, Wattam AR, Xia F, Stevens R. 2014. The SEED and the Rapid Annotation of microbial genomes using Subsystems Technology (RAST). Nucleic Acids Res 42:D206-214.
3. Yu NY, Wagner JR, Laird MR, Melli G, Rey S, Lo R, Dao P, Sahinalp SC, Ester M, Foster LJ, Brinkman FSL. 2010. PSORTb 3.0: improved protein subcellular localization prediction with refined localization subcategories and predictive capabilities for all prokaryotes. Bioinformatics 26:1608-1615.

Table S3. Strains, plasmids and primers used or sequenced as part of this study.

Strain	Origin/Description	Sequencing vendor (platform)[a]	Source[b]
E. gallinarum			
MRx0518	Human isolate	Diversigen (PacBio RS II)	4D Pharma Research Ltd (NCIMB 42488)
MRx0554	Human isolate	GATC (Illumina 2x300 bp paired end)	4D Pharma Research Ltd
MRx0556	Human isolate	GATC (Illumina 2x300 bp paired end)	4D Pharma Research Ltd
MRx1548	Human isolate	MicrobesNG (Illumina 2x250 bp paried end)	4D Pharma Research Ltd
MRx1649	Human isolate	MicrobesNG (Illumina 2x250 bp paried end)	4D Pharma Research Ltd
MRx1650	Human isolate	MicrobesNG (Illumina 2x250 bp paried end)	4D Pharma Research Ltd
MRx1763	Human isolate	MicrobesNG (Illumina 2x250 bp paried end)	4D Pharma Research Ltd
MRx1766	Human isolate	MicrobesNG (Illumina 2x250 bp paried end)	4D Pharma Research Ltd
MRx1775	Human isolate	MicrobesNG (Illumina 2x250 bp paried end)	4D Pharma Research Ltd
MRx1886	Human isolate	MicrobesNG (Illumina 2x250 bp paried end)	4D Pharma Research Ltd
DSM20628	Chicken isolate	MicrobesNG (Illumina 2x250 bp paried end)	DSMZ
DSM20718	Chicken isolate	MicrobesNG (Illumina 2x250 bp paried end)	DSMZ
DSM 100110	Wild mouse isolate	GATC (Illumina 2x300 bp paired end)	DSMZ
DSM28564	Mouse isolate	MicrobesNG (Illumina 2x250 bp paried end)	DSMZ
DSM28565	Mouse isolate	MicrobesNG (Illumina 2x250 bp paried end)	DSMZ
MRx0518 *fliC*::pORI19	Flagellin insertion mutant in MRx0518	n.a.	This study
E. casseliflavus			
MRx0858	Human isolate	GATC (Illumina 2x300 bp paired end)	4D Pharma Research Ltd
DSM25781	Human isolate	MicrobesNG (Illumina 2x250 bp paried end)	DSMZ
DSM7370	Human isolate	MicrobesNG (Illumina 2x250 bp paried end)	DSMZ
E. coli			
E. coli EC101	Cloning host, *repA*$^+$, Kan^R		1
E. coli M15[pREP4]	Cloning host, F-, Φ80Δ*lac*M15, *thi*, *lac*$^-$, *mtl*$^-$, *recA*$^+$, Kan^R		Qiagen

Plasmid	Description	Reference
pORI19	Em^r *repA*$^-$ Ori^+; cloning vector	1
p4D016	507 bp internal fragment *fliC*$_{MRx0518}$ in pORI19	This study
pQE-30	N-terminus 6xHis tag expression vector, Amp^R	Qiagen
p4D013	Full-length *fliC*$_{MRx0518}$ in pQE-30	This study
p4D014	Full-length *fliC*$_{DSM\ 100110}$ in pQE-30	This study

Primer	Description [c]	Sequence (5’ → 3’) [d]	Reference
DC022	FP to clone the full-length *fliC*$_{\mathrm{MRx0518}}$ gene in pQE-30	ACGACGGGATCCATGAGAATCAATACAAACGTTTCAGC	This study
DC023	RP to clone the full-length *fliC*$_{\mathrm{MRx0518}}$ gene in pQE-30	CCCGGGGTCGACTTATCCTTGTAACAAGCTTAATACAC	This study
DC024	FP to clone the full-lenght *fliC*$_{\mathrm{DSM100110}}$ gene in pQE-30	ACGACGGGATCCATGAGAATCAATACAAACGTGTCTGC	This study
DC025	RP to clone the full-length *fliC*$_{\mathrm{DSM100110}}$ gene in pQE-30	CCCGGGGTCGACTTATTGAAGTAAGCTCAATACGCTG	This study
DC020	FP to clone a 507 bp internal fragment of *fliC*$_{\mathrm{MRx0518}}$ in pORI19	CCCGGGGGATCCGCGGTAAATGTTGCTAAAGCATCATCG	This study
DC021	RP to clone a 507 bp internal fragment of *fliC*$_{\mathrm{MRx0518}}$ in pORI19	ACGACGGTCGACCCACAGCATCTTAGGGCGTATGCG	This study
DC047	FP used to amplify the *em* gene on pORI19	CCAAATTAAAGAGGGTTATAATGAACGAG	This study
DC048	RP used to amplify the *em* gene on pORI19	GATGCAGTTTATGCATCCCTTAAC	This study
DC013	FP used to confirm chromosomal insertion of pORI19 in *fliC*$_{\mathrm{MRx0518}}$	CCGATAAATAGTAGCAGAGGGAAACC	This study
DC014	RP used to confirm chromosomal insertion of pORI19 in *fliC*$_{\mathrm{MRx0518}}$	GGCTGAATATCCATCAGAGCTTCCTC	This study
CXCL1-F	Forward qPCR primer to target the human CXCL1 gene	CTGGCTTAGAACAAAGGGGCT	This study
CXCL1-R	Reverse qPCR primer to target the human CXCL1 gene	TAAAGGTAGCCCTTGTTTCCCC	This study
CXCL8-F	Forward qPCR primer to target the human CXCL8 gene	AGCTCTGTGTGAAGGTGCAG	This study

CXCL8-R	Reverse qPCR primer to target the human CXCL8 gene	ATTTCTGTGTTGGCGCAGTG	This study
CCL20-F	Forward qPCR primer to target the human CCL20 gene	CGAATCAGAAGCAGCAAGCAA	This study
CCL20-R	Reverse qPCR primer to target the human CCL20 gene	CCGTGTGAAGCCCACAATAAA	This study
ICAM1-F	Forward qPCR primer to target the human ICAM1 gene	AGCTTCGTGTCCTGTATGGC	This study
ICAM1-R	Reverse qPCR primer to target the human ICAM1 gene	TTTCTGGCCACGTCCAGTTT	This study
NFKBIA-F	Forward qPCR primer to target the human NFKBIA gene	AGGACGAGCTGCCCTATGA	This study
NFKBIA-R	Reverse qPCR primer to target the human NFKBIA gene	GTCCATGTTCTTTCAGCCCCT	This study
TNFAIP3-F	Forward qPCR primer to target the human TNFAIP3 gene	TCAACTGGTGTCGAGAAGTCC	This study
TNFAIP3-R	Reverse qPCR primer to target the human TNFAIP3 gene	ACGCCCCACATGTACTGAGA	This study

[a] n.a.: not applicable. Diversigen Inc. (Houston, TX, USA), GATC Biotech (Konstanz, Germany), MicrobesNG (Birmingham, England, UK).

[b] 4D pharma plc culture collection (https://www.4dpharmaplc.com/developing-science/microrx).

[c] FP: forward primer, RP: reverse primer.

[d] Restriction sites are underlined.

Reference

1. Law J, Buist G, Haandrikman A, Kok J, Venema G, Leenhouts K. 1995. A system to generate chromosomal mutations in *Lactococcus lactis* which allows fast analysis of targeted genes. J Bacteriol 177:7011-7018.
